# The heart’s fibrous web: A bibliometric analysis of cardiac fibrosis in Asia and Oceania

**DOI:** 10.1097/MD.0000000000049017

**Published:** 2026-05-29

**Authors:** Arifah Ahmad Damahuri, Maslinawati Mohamad, Thuhairah Hasrah Abdul Rahman, Vimala RMT Balasubramaniam, Nurdiyana Nasrudin, Nasibah Azme

**Affiliations:** a Laboratory Animal Care Unit, Universiti Teknologi MARA (UiTM), Sungai Buloh, Selangor, Malaysia; b Institute of Medical Molecular Biotechnology, Universiti Teknologi MARA (UiTM), Sungai Buloh, Selangor, Malaysia; c Accounting Research Institute, University Teknologi MARA (UiTM), Shah Alam, Selangor, Malaysia; d Faculty of Medicine, Universiti Teknologi MARA (UiTM), Sungai Buloh, Selangor, Malaysia; e Cardiovascular Advancement Research Excellence Institute (CARE-i), Universiti Teknologi MARA, Malaysia; f Nutrition, Metabolism & Cardiovascular Research Centre, Institute for Medical Research, Ministry of Health Malaysia, Setia Alam, Selangor, Malaysia.

**Keywords:** Asia, cardiac fibrosis, heart fibrosis, Oceania, OpenRefine, VOSviewer

## Abstract

**Background:**

Cardiac fibrosis is a pathophysiologic condition in heart diseases. It refers to an excess deposition of extracellular matrix, which will cause hardening and stiffness in heart tissues, leading to heart failure. By considering the high prevalence of cardiac fibrosis globally, a bibliometric analysis is conducted to elucidate the research trend involving the unique perspectives in both the Asia and Oceania regions.

**Methods:**

Data were retrieved from the Scopus database without restrictions on publication year. Documents related to cardiac fibrosis affiliated with countries in Asia and Oceania were identified using the United Nations geoscheme classifications. The dataset was curated using biblioMagika and OpenRefine. Then, bibliometric mapping was conducted using VOSviewer to analyze co-authorship and keyword co-occurrence networks.

**Results:**

A total of 983 publications were identified from 1985 to 2025, with a marked increase in research output from 2010 onwards. China emerged as the leading contributor in terms of productivity and total citations, while Australia showed the highest citation impact per publication. High-impact institutions and authors were identified, and core themes included molecular mechanisms such as transforming growth factor beta signaling, extracellular matrix remodeling, and noncoding RNAs.

**Conclusion:**

This study is the first to systematically examine cardiac fibrosis research output across Asia and Oceania using bibliometric methods. It contributes novel insights into the regional dynamics, intellectual structure, and emerging priorities within the field. By identifying key actors, collaboration networks, and knowledge gaps, this research provides valuable guidance for scholars, funding agencies, and policymakers to advance cardiac fibrosis research in regional and global contexts. However, reliance on citation-based metrics may introduce temporal bias, as older publications tend to accumulate more citations over time. Future studies should employ multi-database strategies, altmetric indicators, and machine learning approaches to address these limitations and capture a broader spectrum of research impact.

## 1. Introduction

Fibrotic diseases cause annually more than 800,000 deaths worldwide, with the majority accounting for lung and cardiac fibrosis.^[1]^ Cardiac fibrosis is a pathological occurrence characterized by the abnormal accumulation of extracellular matrix (ECM) proteins within the myocardium, leading to impaired myocardial functioning and augmented stiffness, which may indicate the initiation of reparative or maladaptive mechanisms. Although structural collagen is indispensable for upholding physiological cardiac operation, fibrosis signifies pathological modifications corresponding to deteriorated clinical consequences. It serves as a prevalent course in chronic myocardial disease and correlates with heart failure.

Asia, the most populous continent globally, comprises approximately 4.68 billion individuals, representing roughly 59.5% of the world population as of 2021.^[2]^ Westhuysen reported that China, with an impressive population of 1.5 billion, is Asia’s most extensive nation.^[3]^ In contrast, Oceania, as a continent, exhibits a considerably smaller human population, where Australia serves as the largest and most densely inhabited country within this region. Recent estimations for the year 2023 project a population of approximately 26,439,111 in Oceania.^[4]^ Furthermore, cardiac fibrosis presents a considerable concern in the regions of Asia and Oceania, as heart failure impacts an estimated 64.3 million people worldwide in the year 2017.^[5]^ Comparatively, the incidence of concomitant diseases and multiple chronic conditions is more pronounced among Asian heart failure patients in contrast to their Western counterparts. With the increasing cardiac fibrosis burden, especially in Asia and Oceania, the research focus is shifting not only to provide clinical and molecular mechanistic insights into this disease but also to propose new technologies that could expand recognition and knowledge of the disease.

While the biological and clinical consequences of cardiac fibrosis have been extensively studied, recent advancements in computational cardiology have introduced machine learning, particularly deep learning and network-based approaches, to predict and model cardiac conditions. For example, Surucu et al^[6]^ applied convolutional neural networks to predict the onset of paroxysmal atrial fibrillation, and Isler et al^[7]^ used heart rate variability in a multi-stage classification model for diagnosing heart failure. More recently, artificial intelligence has been applied to fibrosis-specific contexts, including the detection of cardiac fibrosis from electrocardiographic signals^[8]^ and the use of logic-based machine learning to examine fibroblast behavior in response to drugs.^[9]^ The studies contribute to the evolution of the functions that the computational methods could gain by supporting traditional research, whereas the possible utilization of this educational area regarding cardiac fibrosis of the Asian and Oceania regions is under-researched. On a larger scale of biology, Gosak et al^[10]^ surveyed the role of network structures in the morphological and functional organization of living systems, generating a conceptual application in cardiac remodeling. These advances provide further insight into the changing state of cardiac fibrosis research, such as new directions likely to define future studies.

Bibliometric analysis is a set of statistical methods used to analyze the processes of generation and dissemination of information and literature review, with a focus on books, articles, and other publications, specifically on scientific material.^[11,12]^ This methodology is traditionally used in the fields of library and information science, and is closely intertwined with scientometrics and the management of scientific metrics and indicators. The use of bibliometric analysis allows for the revealing of basic areas of research or of authors and the relationships that link them. Therefore, the point of view of the bibliometric analysis in this study was to assess trends in the growth of burden in cardiac fibrosis in Asian and Oceanian populations, emphasizing the need for more research and intervention strategies. The following are the research questions that denote the aim of this bibliometric study:

1. What are the publication trends in the field of cardiac fibrosis in Asia and Oceania, and how have they changed over time?
2. Who are the most prolific authors in cardiac fibrosis in Asia and Oceania, and what are the key themes and topics in their research?
3. Which key players, organizations, and countries have contributed to developing cardiac fibrosis publications in Asia and Oceania?
4. What are the most highly cited documents in the field of cardiac fibrosis, and what are the key themes and topics they address?
5. What are the most common keywords in the literature on cardiac fibrosis in Asia and Oceania, and how have they evolved?

## 2. Literature review

The significance of research conducted in Asia and Oceania is considerable for various factors. Through the application of cultural context analysis, researchers can gain an understanding of the specific cultural setting in which their research is situated. Additionally, such investigations may yield insights into human behavior, social issues, and research approaches. The presence of diverse cultures, languages, and lifestyles in Asia and Oceania contributes to enriching research and product development by fostering a more comprehensive understanding of various aspects.^[13]^ Additionally, research conducted in these regions contributes to resolving regional problems and obstacles, including social issues, misconduct, and cultural differences.

Next, taking part in research based in Asia and the Oceania region promotes collaboration among researchers, both countries and cultures, eventually producing new knowledge and discoveries. Conducting research in these areas creates opportunities for researchers to tackle methodological and ethical dilemmas, such as getting access to study participants, dealing with language barriers, and ensuring the informed consent of participants. Importantly, research conducted in Asia and Oceania is essential for advancing knowledge and collaboration in various domains of study. It is necessary to see it as a key part of the research undertaken on a global scale.

While the field of cardiac fibrosis has been the subject of numerous bibliometric analyses, not all of them specifically concentrate on countries in Asia and the Oceans. The main outcomes covered biomarkers of cardiac fibrosis, its pathophysiological mechanisms, treatment strategies, and related pathologies. Considering the prevalence of cardiac fibrosis in these regions, this study will include all data related to both regions. Despite the commonly used interchangeable words cardiac fibrosis and heart fibrosis, they may imply minor differences concerning research findings. It can be the contextual appropriateness, stylistic diversity, or language dynamics. However, both terms provide important insights into the global research landscape of cardiac fibrosis, which could be beneficial in addressing the context in the countries of Asia and Oceania. The key findings of these studies form the basis for further research in Asia and Oceania.

## 3. Methods

A comprehensive bibliometric analysis was conducted to evaluate the research landscape on cardiac fibrosis, specifically within the geographic scope of Asia and Oceania. The regional classification for Asia and Oceania used in this study is based on the United Nations Statistics Division’s M49 regional classification geoscheme,^[14]^ an internationally recognized framework that groups countries by macro-geographical (continental) regions and subregions for statistical purposes. As per this classification, Asia includes 5 subregions: Central, Eastern, South-Eastern, Southern, and Western Asia, while Oceania includes Australia and New Zealand, Melanesia, and Micronesia. The complete list of countries included under each subregion is presented in Appendix A. The Scopus database was selected as the primary data source owing to its extensive coverage of peer-reviewed literature across disciplines. The metadata used in this study were retrieved in April 2025, covering the period from 1985 to April 2025. It contained information such as the author’s full name, subject area, document type, source type, language, country, affiliation, citation data, keywords, and references.

### 3.1. Search strategy and data collection

**Figure 1.**
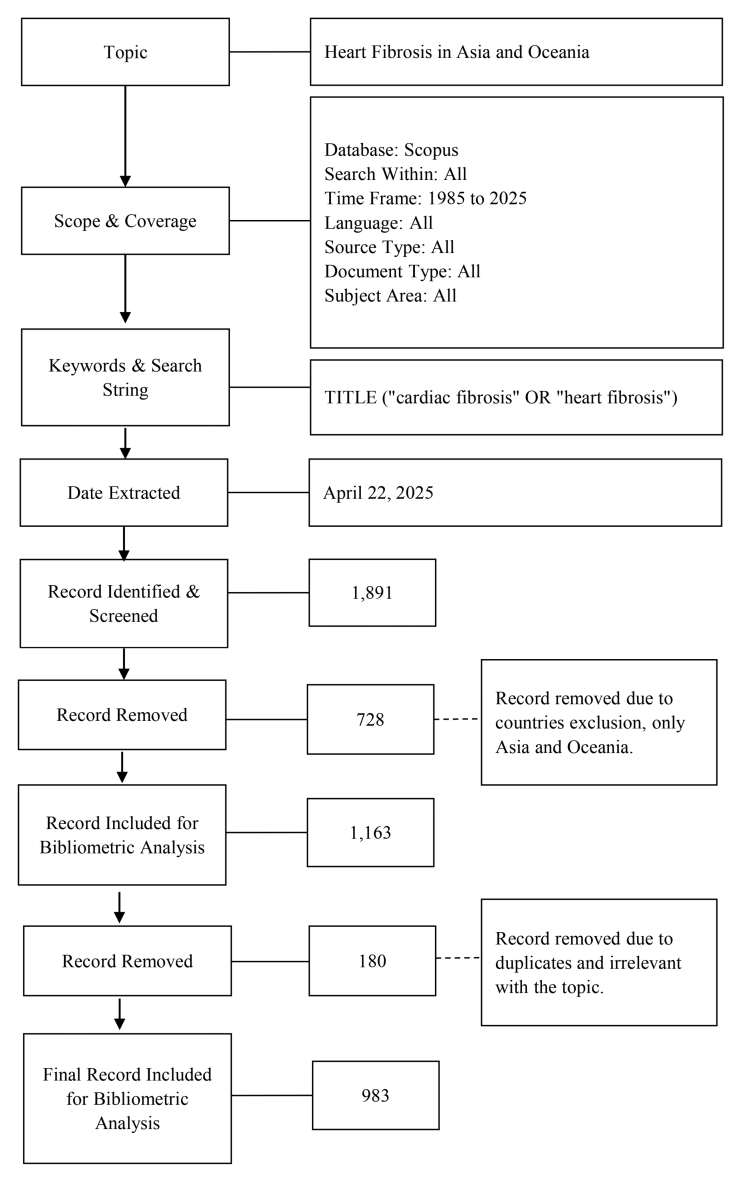
Flow diagram of the search strategy. Out of 1891 records identified, 983 relevant publications on cardiac fibrosis in Asia and Oceania were included after screening and exclusion steps.

The search included all types of publication titles containing the keyword “cardiac fibrosis” or “heart fibrosis” by executing the search string within TITLE. We included publications available in all languages, with no restrictions imposed on time frame, source type, or document type, which yielded 1891 publications. Then, the data was constrained to Asia and Oceania countries defined by the United Nations geoscheme, which narrowed down to 1163 publications. The dataset’s titles, abstracts, and keywords were examined. In cases of ambiguity, the entire text was read. This resulted in excluding errata, retracted, corrigenda, correction notices, and duplicated documents. Subsequently, 983 publications were downloaded for analysis. The process was accomplished by 2 well-trained researchers to ensure the reliability and validity of the data. Figure 1 provides a graphic representation of the bibliometric search strategy, which entailed the selection of topics and scoping of interests, followed by keyword search in Scopus and subsequently elimination of nonrelevant or duplicate records sequentially, resulting in only 983 records that were included in the final bibliometric analysis

### 3.2. Tools and data analysis

Analysis of the included studies was performed through Microsoft Excel 2023, biblioMagika,^[15]^ OpenRefine, and VOSviewer.^[16]^ The first organization of the data was performed in Microsoft Excel. Consequently, biblioMagika converted the Scopus metadata, and OpenRefine was then used subsequently to clean up and standardize the names of authors, affiliations, and keywords.^[17]^ The network analysis visualization of data cleaning and harmonization was performed in VOSviewer as a graphic interface. The bibliometric maps and the network-based image visualizations, such as the co-authorship and co-occurrence analysis, were then generated using VOSviewer. Importantly, this tool also points out the evolution of research themes and topics across the cardiac fibrosis publications.

## 4. Results

### 4.1. Documents profiles

Table 1 shows the descriptive statistics of the citation performance and author productivity metrics of the dataset, including 983 publications on cardiac fibrosis in Asia and Oceania between the years 1985 and 2025. The dataset has received 29,734 citations, which reflect the cumulative scholarly impact of the publications presented in the dataset. The papers obtained 30.25 citations on average, implying moderate to high visibility and impact in the scientific community. When considering only the 897 papers that have been cited at least once, the average citation rises to 33.15 citations per cited paper, indicating that most of the cited literature attained substantial recognition. The citation rate was 743.35 per year, proving the relevancy of the field and active scholastic engagement throughout the 41 years of the publication. This gradual accumulation of citations can be suggestive of a sustained scientific and clinical interest in cardiac fibrosis in the Asia and Oceania region.

**Table 1:** Citation metrics.

Information	Data
Publication years	1985–2025
Total publications	983
Citable year	41
Number of contributing authors	983
Number of cited papers	897
Total citations	29,734
Citation per paper	30.25
Citation per cited paper	33.15
Citation per year	743.35
Citation per author	30.25
Author per paper	1.00
Citation sum within *h*-core	24,408
*h*-index	82
*g*-index	123
*m*-index	2.000

Source: Generated by the author(s) using biblioMagika (Ahmi^[15]^).

The 983 contributing authors were equal to the number of publications, which yielded an author-to-paper of 1.00. Although unusual for a biomedical bibliometric profile, where multi-authorship is the norm, such an indicator may indicate disambiguation of author names or inclusion of single-authored or institutionally affiliated works. However, the citation per author, which is also 30.25, reflects author contribution on papers, strengthening the direct relationship between author contribution and scholarly impact in this dataset. The data produced an *h*-index of 82, which means that 82 articles were cited at least 82 times each. This gives a strong indication of long-term academic impact because a large *h*-index suggests not only productivity but also steady performance in terms of citations. Moreover, the *g*-index of 123 highlights the existence of highly cited publications in the database; the *g*-index is more reflective of outlier-dense publications in the dataset and records more of the citation ecosystem. Furthermore, the *g*-index of 123 underscores the presence of highly cited publications within the dataset; the *g*-index has a higher weight on citation-intensive works, therefore, covering the entire citation environment more accurately.

The *m*-index is the *h*-index divided by the number of active publication years (*h*-index/citable years). The *m*-index was 2.000. This value indicates a high magnitude of sustained impact each year, which is essential when comparing across disciplines or geographic locations where research maturity may vary. Finally, the sum of citations at the *h*-core ended up being 24,408, with the top 82 articles corresponding to about 82.1 percent of all citations. The observed finding reflects the imbalance of a limited number of very influential studies, which is typical of biomedical bibliometrics, consistent with the Lotka law Matthew effect in scientific publishing.

Table 2 outlines the distribution of document types among the 983 records included in this bibliometric analysis of cardiac fibrosis research in Asia and Oceania. The vast majority of the publications (n = 855; 86.98%) were categorized as original research articles, indicating that empirical investigations constitute the primary mode of knowledge production in this field. This predominance aligns with the nature of biomedical and clinical sciences, where data-driven studies form the foundation for advancing mechanistic insights, diagnostic techniques, and therapeutic strategies related to cardiac fibrosis. A total of 89 review articles (9.05%) were identified, reflecting a moderate emphasis on synthesis-oriented scholarship. The presence of nearly one-tenth of the documents as reviews suggests an established and maturing research field, where the consolidation of evidence and the development of conceptual frameworks have become integral to scholarly discourse.

**Table 2:** Document type.

Document type	Total publications	Percentage (%)
Article	855	86.98%
Review	89	9.05%
Letter	16	1.63%
Conference paper	8	0.81%
Note	7	0.71%
Editorial	4	0.41%
Short survey	3	0.31%
Book chapter	1	0.10%

Source: Generated by the author(s) using biblioMagika (Ahmi^[15]^).

Other publication formats, including letters (n = 16; 1.63%), conference papers (n = 8; 0.81%), notes (n = 7; 0.71%), and editorials (n = 4; 0.41%), collectively accounted for a minor portion of the total output. These document types typically serve complementary functions, such as preliminary findings, scholarly commentary, or community engagement, and are indicative of ongoing dialogs and early-stage research dissemination. Additionally, short surveys (n = 3; 0.31%) and a single book chapter (n = 1; 0.10%) were also retrieved. Their relatively low frequency underscores the limited role of non-journal formats and alternative scholarly outputs in this field, possibly reflecting the strong preference for peer-reviewed journal articles in biomedical sciences.

The distribution pattern observed in Table 2 is characteristic of a field driven by experimental and translational research. The high proportion of original research articles suggests robust academic activity focused on generating novel findings, while the presence of review papers points to efforts at theoretical consolidation. The marginal contribution of other formats is consistent with publication norms in medical sciences, where impact and visibility are predominantly associated with peer-reviewed articles.

Table 3 presents the distribution of source types for the 983 publications analyzed in this study on cardiac fibrosis within the Asia–Oceania region. The data reveal a near-total dominance of journal-based publications, with 980 of 983 documents (99.69%) disseminated through peer-reviewed academic journals

**Table 3:** Source type.

Source type	Total publications	Percentage (%)
Journal	980	99.69%
Book Series	2	0.20%
Conference Proceeding	1	0.10%

Source: Generated by the author(s) using biblioMagika (Ahmi^[15]^).

The overwhelming concentration of publications in journals (99.69%) underscores the central role of journal articles as the primary medium for scholarly communication in biomedical and clinical sciences. This pattern is consistent with global norms in cardiovascular and fibrosis research, where journals serve as the principal platforms for disseminating both original research and systematic reviews. Journals are also more likely to be indexed, peer-reviewed, and widely accessible, which enhances both the credibility and visibility of published work. As such, this dominance directly influences citation patterns and research visibility, as journal articles tend to accrue more citations due to broader circulation and indexing in citation databases such as Scopus.

In contrast, book series (n = 2; 0.20%) and conference proceedings (n = 1; 0.10%) collectively account for <0.5% of the total output. These marginal figures suggest that cardiac fibrosis research in this geographic context is not commonly disseminated through edited volumes or scientific meetings. This may reflect a discipline-specific preference for journals, or possibly the lack of regional or topic-specific conference series that publish proceedings indexed in Scopus. It is also worth noting that conference proceedings, although limited in number, may represent preliminary findings or early-stage research. While such outputs typically receive fewer citations and lower scholarly visibility, they can be indicative of emerging research directions, particularly in translational science or biomedical engineering contexts related to fibrosis.

Table 4 presents the linguistic distribution of the 983 publications on cardiac fibrosis research from Asia and Oceania. The data indicate a strong dominance of English-language publications, with minimal contributions from other regional languages. A total of 936 publications (95.22%) were written in English, reflecting the global predominance of English as the lingua franca of scientific communication. This trend is particularly pronounced in biomedical research, where English-language journals dominate international indexing databases such as Scopus, PubMed, and Web of Science. The overwhelming use of English in the present dataset suggests that researchers in Asia and Oceania are engaging with a global scholarly audience and prioritizing visibility in high-impact, internationally accessible journals.

**Table 4:** Languages.

Language	Total publications	Percentage (%)
English	936	95.22%
Chinese	44	4.48%
Persian	2	0.20%
Japanese	1	0.10%

Source: Generated by the author(s) using biblioMagika (Ahmi^[15]^).

Despite the predominance of English, a small proportion of publications were disseminated in Chinese (n = 44; 4.48%), followed by Persian (n = 2; 0.20%), and Japanese (n = 1; 0.10%). The presence of Chinese-language publications, although limited, is notable and reflects the strong domestic research infrastructure in China and Chinese-speaking regions. These documents may be published in national journals not widely indexed in global databases, which could influence their citation performance and international visibility. Besides, the negligible contributions from Persian and Japanese indicate that, while cardiac fibrosis research is conducted in countries where these languages are spoken (e.g., Iran and Japan), such work is predominantly published in English to enhance scholarly reach and impact.

Table 5 outlines the disciplinary landscape of cardiac fibrosis research within Asia and Oceania, as indexed by Scopus subject categories. The findings highlight the multidisciplinary nature of the field, with notable concentrations in biomedical and life sciences.

**Table 5:** Subject area.

Subject area	Total publications	Percentage (%)
Medicine	503	51.17%
Biochemistry, Genetics and Molecular Biology	478	48.63%
Pharmacology, Toxicology and Pharmaceutics	284	28.89%
Immunology and Microbiology	71	7.22%
Chemistry	43	4.37%
Multidisciplinary	42	4.27%
Chemical Engineering	31	3.15%
Neuroscience	24	2.44%
Agricultural and Biological Sciences	23	2.34%
Environmental Science	22	2.24%
Engineering	20	2.03%
Computer Science	19	1.93%
Materials Science	16	1.63%
Physics and Astronomy	13	1.32%

Source: Generated by the author(s) using biblioMagika (Ahmi^[15]^).

The majority of publications were classified under Medicine (n = 503; 51.17%), followed closely by Biochemistry, Genetics and Molecular Biology (n = 478; 48.63%), and Pharmacology, Toxicology, and Pharmaceutics (n = 284; 28.89%). These 3 domains represent the core scientific pillars of cardiac fibrosis research. The dominance of Medicine reflects the clinical focus of cardiac fibrosis studies, which encompass diagnosis, pathophysiology, imaging, and therapeutic strategies related to fibrotic remodeling of the myocardium. Meanwhile, the high proportion of publications in Biochemistry, Genetics, and Molecular Biology suggests a strong emphasis on molecular mechanisms such as transforming growth factor beta (TGF-β) signaling, ECM remodeling, fibroblast activation, and gene expression profiling, which are central to understanding fibrogenesis. Then, the significant representation in Pharmacology, Toxicology and Pharmaceutics underscores ongoing efforts to develop anti-fibrotic drugs, evaluate pharmacodynamics, and explore repurposing of existing therapeutics. These results affirm that cardiac fibrosis research in the region is firmly rooted in translational science, bridging basic molecular insights with clinical applications.

Beyond the biomedical core, several other subject areas, though quantitatively smaller, reflect the interdisciplinary expansion of the field. First, Immunology and Microbiology (7.22%) points to the growing recognition of immune pathways (e.g., macrophage polarization, cytokine signaling) in cardiac fibrotic processes. Second, Chemistry (4.37%), Chemical Engineering (3.15%), and Materials Science (1.63%) likely contribute through research in biomaterials, drug delivery systems, or chemical probes for imaging fibrosis.

Third, the presence of Environmental Science (2.24%) and Agricultural and Biological Sciences (2.34%) may relate to studies exploring environmental cardiotoxins, natural compounds, or animal models of fibrosis. Fourth, Computer Science (1.93%) and Engineering (2.03%) likely reflect advancements in bioinformatics, systems biology, and computational modeling, such as predictive modeling of fibrosis progression or machine learning approaches for image-based diagnosis. Of particular interest is the inclusion of multidisciplinary publications (4.27%), indicating the convergence of multiple domains in the study of cardiac fibrosis. These entries may represent integrative reviews, collaborative efforts, or publications in journals with a broad scope.

### 4.2. Publication trends

Table 6 presents a comprehensive longitudinal overview of the annual publication output, citation performance, and bibliometric impact indicators of cardiac fibrosis research from Asia and Oceania between 1985 and 2025. The dataset reveals a distinct evolution in the field’s development, transitioning from a nascent research area in the 1980s and 1990s to a phase of exponential growth in publication output and scholarly influence from the mid-2000s onward.

**Table 6:** Publication by year.

Year	TP	NCA	NCP	TC	C/P	C/CP	*h*	*g*	*m*
1985	1	1	1	8	8.00	8.00	1	1	0.024
1987	1	1	1	9	9.00	9.00	1	1	0.026
1992	1	1	0	0	0.00	0.00	0	0	0.000
1994	2	2	2	555	277.50	277.50	2	2	0.063
1995	3	3	3	280	93.33	93.33	3	3	0.097
1996	1	1	1	100	100.00	100.00	1	1	0.033
1997	1	1	1	49	49.00	49.00	1	1	0.034
1998	2	2	2	197	98.50	98.50	2	2	0.071
2000	2	2	2	567	283.50	283.50	2	2	0.077
2001	2	2	2	129	64.50	64.50	2	2	0.080
2002	4	4	3	182	45.50	60.67	3	4	0.125
2003	6	6	6	588	98.00	98.00	6	6	0.261
2004	4	4	4	197	49.25	49.25	3	4	0.136
2005	4	4	4	337	84.25	84.25	4	4	0.190
2006	6	6	6	267	44.50	44.50	5	6	0.250
2007	5	5	4	124	24.80	31.00	3	5	0.158
2008	11	11	11	727	66.09	66.09	9	11	0.500
2009	9	9	9	425	47.22	47.22	8	9	0.471
2010	15	15	14	751	50.07	53.64	11	15	0.688
2011	11	11	11	513	46.64	46.64	9	11	0.600
2012	24	24	23	1901	79.21	82.65	16	24	1.143
2013	21	21	21	1112	52.95	52.95	17	21	1.308
2014	27	27	26	1293	47.89	49.73	17	27	1.417
2015	36	36	35	1671	46.42	47.74	25	36	2.273
2016	44	44	42	1472	33.45	35.05	24	37	2.400
2017	62	62	62	3137	50.60	50.60	33	55	3.667
2018	58	58	57	2164	37.31	37.96	28	45	3.500
2019	66	66	66	2788	42.24	42.24	33	51	4.714
2020	70	70	69	2016	28.80	29.22	27	41	4.500
2021	92	92	90	2760	30.00	30.67	30	47	6.000
2022	101	101	98	1657	16.41	16.91	23	32	5.750
2023	107	107	99	1187	11.09	11.99	19	27	6.333
2024	118	118	98	531	4.50	5.42	12	15	6.000
2025	66	66	24	40	0.61	1.67	3	3	3.000
Total	983	983	897	29,734	30.25	33.15	82	123	2.000

C/CP = average citations per cited publication, C/P = average citations per publication, *g* = *g*-index, *h* = *h*-index, *m* = *m*-index, NCA = number of contributing authors; NCP = number of cited publications, TC = total citations, TP = total number of publications.

Source: Generated by the author(s) using biblioMagika (Ahmi^[15]^).

Overall, the first 15 years of the study period (1985–2000) documented minimal publication output with fewer than 5 publications annually. This time could be considered the pioneering phase in the study of cardiac fibrosis in Asia and Oceania, as; preliminary, exploratory manuscripts were written during this time, and initial mechanistic hypotheses were formed. Although the publications are few, their average citation frequencies of papers during this period are quite high. As an example, in 1994 and 2000, the average citation counts in some publications were 277.5 and 283.5, respectively. These statistics imply that a few trailblazing studies were instrumental to the field and became seminal works referenced in future works. Some of these early contributions could be the basic investigation into the mechanism of fibrosis, the histological description of fibrosis, or models of fibrosis, which still form the basis of current research.

An increased research productivity can be identified between 2001 and 2010. The number of publications per year used to be 2 to 6 publications in the early 2000s and 15 by 2010. Citation performance was steady throughout this decade, with average citation per paper being above 40 in the majority of years and above 60 in a number of cases. It implies an increasing amount of research activity and an improvement in the quality and relevance of the output. Bibliometric indicators (e.g., *h*-index and *g*-index) also start to grow with a more consistent rate at this stage, quantifying the productivity and a continued active interest in citation. This time could merge with the solidification of research methods into molecular research, the assimilation of new imaging technology, and the development of pharmacological research into fibrotic pathways.

The years 2011 to 2019 constitute a time of accumulated growth in the number of publications. The number of publications per year rose almost sixfold between 2011 (11 publications) and 2019 (66 publications). This increase in production is complemented by strong citation metrics. In 2019, the total annual citation count was 2788, and the mean citation count per publication was over 40, which shows not only a quantitative but also a high academic impact. In addition to this, bibliometric indicators, like the *h*-index and *g*-index, display dramatic jumps in this timeframe (e.g., *h*-index increased by 24–33 between 2011 and 2019), demonstrating a maturing research environment with increasing numbers of authors publishing high-quality and highly cited articles. The *m*-index, a normalization of the *h*-index to years since first publication, also increases notably during this period, to a maximum of 4.714 in 2019. This acceleration probably marks a combination of factors, such as increased regional investment into biomedical research, international collaboration, and increased clinical interest in cardiomyopathic fibrosis.

From 2020 onward, there is a further increase in publication volume, peaking at 118 publications in 2024. However, average citation metrics begin to decline during this period. For instance, the average citations per paper decreased from 42.24 in 2019 to 11.09 in 2023, and to 4.50 in 2024. This decline is not necessarily indicative of reduced research quality but may be attributed to the recency of publication and insufficient time for citations to accumulate. The citation lag effect is a well-recognized phenomenon in bibliometrics, whereby newly published papers have not yet had the opportunity to be cited. As a result, citation-based metrics for recent years tend to underestimate long-term impact. The relatively low *m*-index values for the final years further corroborate this interpretation. In addition, the coronavirus disease 2019 pandemic had both disruptive and stimulatory effects on cardiac research. While many laboratories faced operational constraints, the discovery of severe acute respiratory syndrome coronavirus 2-related myocardial injury and post-viral fibrosis prompted a surge in related publications. In 2025, the number of publications sharply declined to 66, possibly reflecting incomplete data for the current year at the time of analysis. Additionally, the number of cited papers drops to 24, with only 40 citations in total, suggesting that many of these articles are yet to be indexed or cited. Despite this, a modest *h*-index and *g*-index of 3 have already been recorded, which may increase as these articles mature in the literature.

Across the 41-year study period, a total of 983 publications were produced by an equal number of contributing authors as reflecting the cumulative performance and research trajectory. Of these, 897 publications (91.2%) received at least 1 citation, resulting in a total citation count of 29,734. The overall average citation per publication stands at 30.25, and the citation per cited publication is 33.15, reflecting strong citation performance for a field within a geographically defined scope. The cumulative *h*-index of 82, *g*-index of 123, and *m*-index of 2.000 provide further evidence of the field’s growing academic maturity and sustained research impact.

The data in Table 6 highlight a clear trajectory of scholarly development: from sporadic early contributions to a period of consolidation and growth in the 2000s, followed by a phase of robust productivity and rising influence in the past decade. This temporal pattern reflects the increasing scientific, clinical, and therapeutic relevance of cardiac fibrosis as a research priority across Asia and Oceania. It also reveals that the field has transitioned from a peripheral scientific interest to becoming a key domain of translational cardiovascular research with strong international integration.

**Figure 2.**
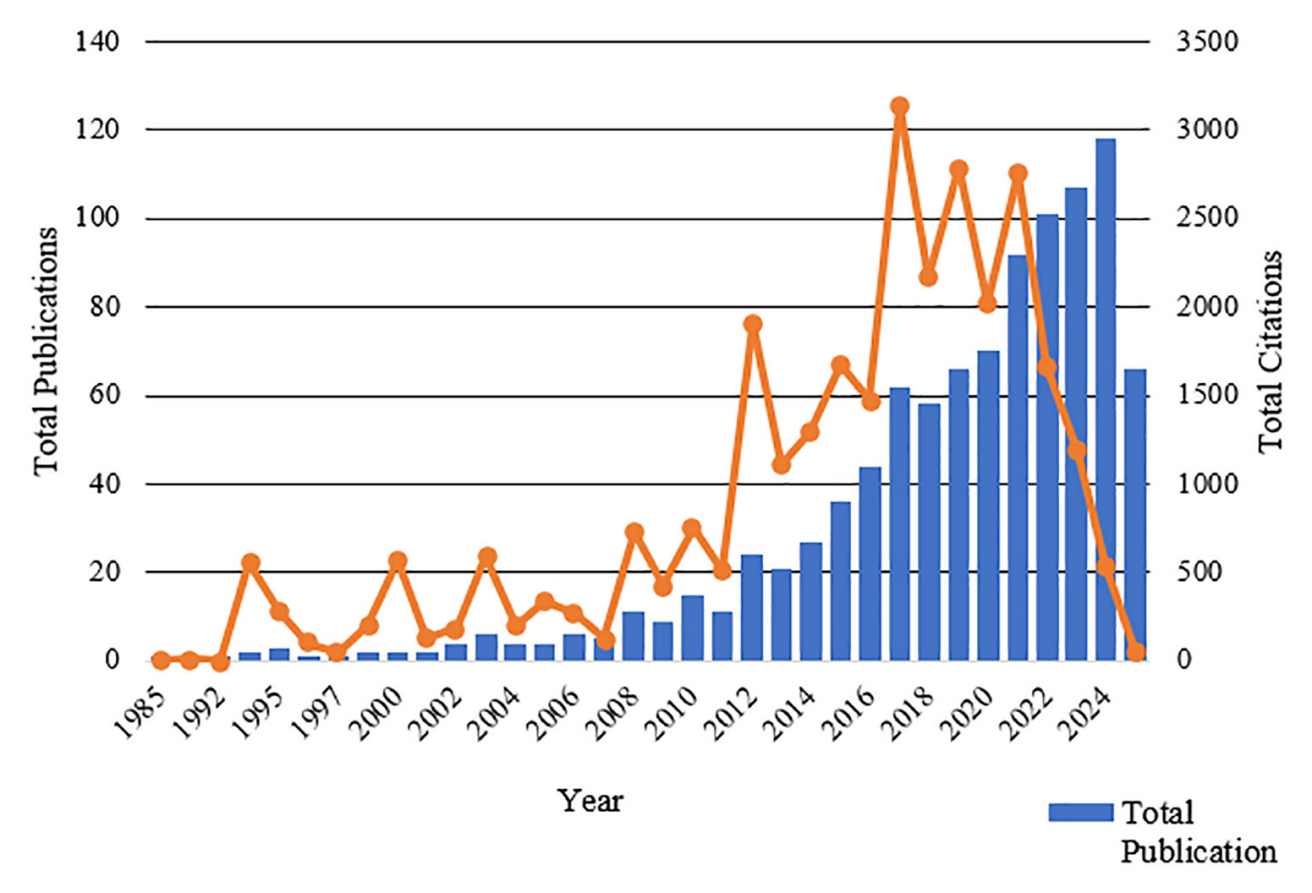
Total publications and citations by year. The figure shows a steady rise in publications on cardiac fibrosis in Asia and Oceania from 1985 to 2025, with a notable increase after 2010. Citations peaked in 2018, reflecting growing impact and interest in the field. Source: Generated by the author(s) using biblioMagika (Ahmi^[15]^).

**Figure 3.**
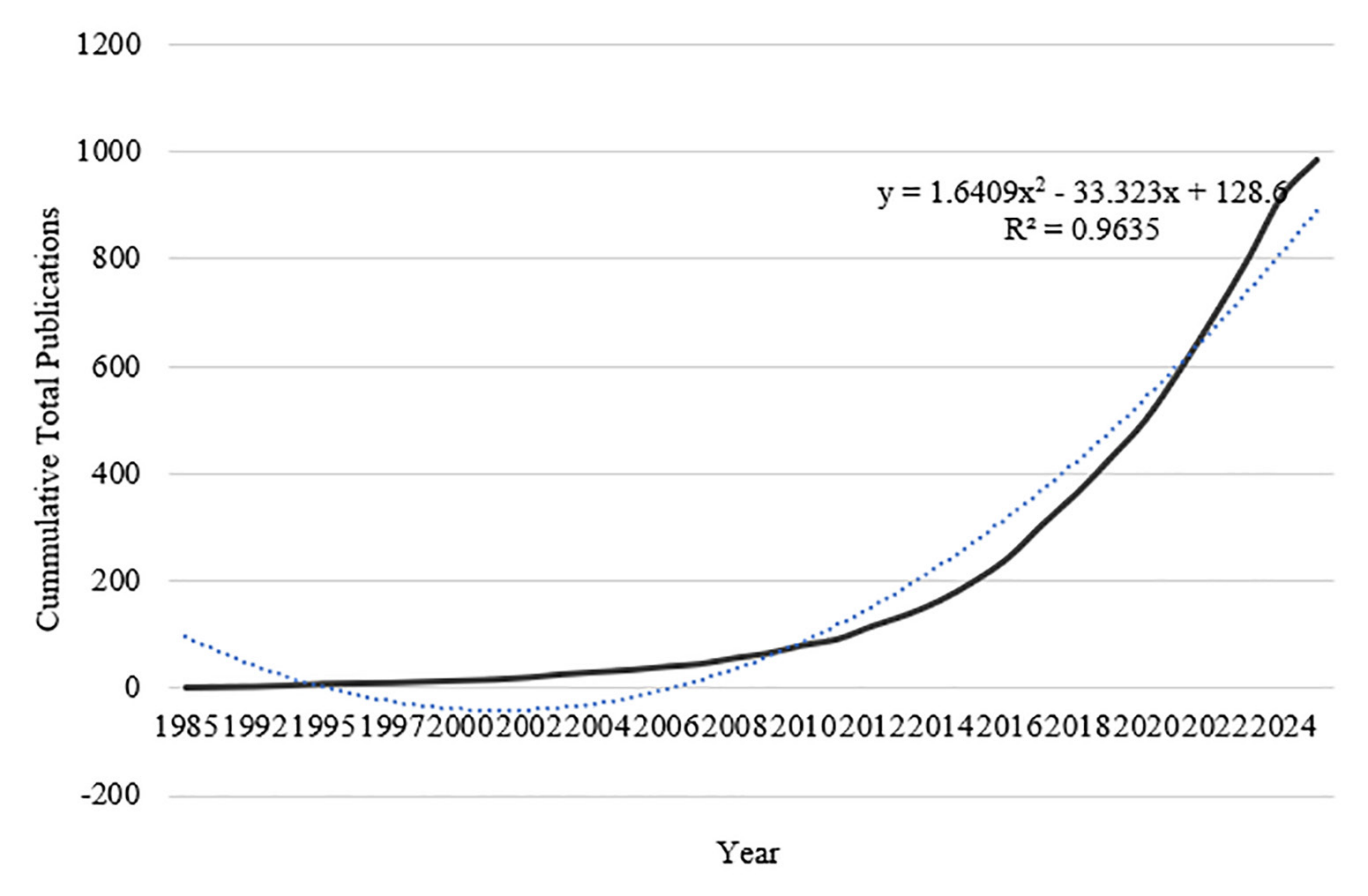
Publication growth. Cumulative publications on cardiac fibrosis in Asia and Oceania show a strong upward trend, with rapid growth after 2010. The fitted curve (*R*^2^ = 0.9635) indicates consistent acceleration in research output. Source: Generated by the author(s) using biblioMagika (Ahmi^[15]^).

Figures 2 and 3 comprehensively depict the temporal evolution and scholarly impact of cardiac fibrosis research within Asia and Oceania, highlighting both the volume of scientific output and its intellectual influence over time. Figure 2 shows the annual number of publications (blue bars) and citations (orange line) from 1985 to 2025, while Figure 3 presents the cumulative growth in publications over the same period, modeled using a second-degree polynomial curve. Collectively, these figures underscore 3 distinct growth phases. First is the emergent phase (1985–2005). This initial period is characterized by a low but steady number of annual publications, with minimal citation activity. Both figures reflect limited research engagement and awareness of cardiac fibrosis as a focal scientific concern. The cumulative publication curve in Figure 3 is nearly flat during this phase.

The phase from 2006 to 2008 illustrates an acceleration phase, whereby a noticeable increase in annual publications and citations occurs (Fig. 2), coinciding with the first clear upward inflection in the cumulative curve in Figure 3. This period likely reflects rising global attention to heart failure with preserved ejection fraction, a condition in which cardiac fibrosis plays a key mechanistic role, and advancements in molecular and imaging technologies, such as cardiac MRI, transcriptomics, and fibrosis biomarkers. Regional investment in biomedical research and the rise of collaborative networks in the Asia-Pacific may have also catalyzed output growth.

The exponential expansion phase occurs between 2016 and 2024. This is the most prolific period, as evidenced by the steep rise in both annual publications and citations in Figure 2 and the sharply increasing slope of the cumulative curve in Figure 3. Notably, citations peak around 2019 to 2020, suggesting that influential works were published shortly before or during this time. This acceleration aligns with increased translational research, drug discovery efforts targeting fibrotic pathways, and heightened global awareness of fibrosis-related pathophysiology amid broader cardiovascular disease trends.

The citation trajectory in Figure 2 reveals a marked increase during the same period when publication output intensified. The lag between the rise in publications and the citation peak suggests a typical bibliometric latency, where influential articles accumulate citations over several years post-publication. The decline in citations after 2020 may reflect incomplete indexing for the most recent years or a time-lag artifact rather than an actual decline in impact.

Figure 3’s polynomial regression curve (*R*^2^ = 0.9635) provides a robust statistical model of publication growth, confirming a high degree of predictability and continuity in the expansion of the field. The quadratic term signifies a nonlinear, accelerating trajectory, suggesting that the research domain is still in a growth phase with no evident saturation point. This pattern implies sustained scholarly interest, likely driven by the clinical burden of fibrosis-driven cardiac diseases or multidisciplinary convergence involving cardiology, molecular biology, pharmacology, and data science. Besides, growing institutional capacity and international collaboration across the Asia–Oceania region are possibly one of the factors.

The combination of Figures 2 and 3 reveals a compelling and coherent narrative. Over the past ten years, cardiac fibrosis research in Asia and Oceania has developed from relative obscurity into a fast-growing sector of high scientific and clinical value. The rapid increase in both annual publication and citation counts, together with the strong polynomial fit of cumulative growth, indicates not just a quantitative expansion of the research ecosystem, but also qualitative development. Such trends point to an active field that will continue to be innovative and contribute globally in the future.

### 4.3. Publication by authors

Table 7 shows the ranking of the 25 most productive authors ranked by their total number of publications (TP) in the area of cardiac fibrosis. It depicts a more refined picture by incorporating other metrics, including citation, impact frequencies, and consistency of publication.

**Table 7:** Most productive authors.

Author’s name	Current affiliation	Country	TP	NCP	TC	C/P	C/CP	*h*	*g*	*m*
Tao, Hui	Anhui Medical University	China	30	30	928	30.93	30.93	17	30	1.308
Yang, Jing Jing	Anhui Medical University	China	27	26	724	26.81	27.85	15	26	1.154
Tang, Qi Zhu	Wuhan University	China	22	21	1073	48.77	51.10	14	22	1.273
Shi, Kai Hu	Nanjing University of Chinese Medicine	China	17	16	633	37.24	39.56	14	17	1.077
Zhao, Jian Yuan	Shanghai Jiao Tong University	China	15	15	326	21.73	21.73	8	15	1.600
Liu, Zhi Yan	Anhui Medical University	China	14	14	185	13.21	13.21	7	13	2.333
Lin, Li Chan	Anhui Medical University	China	14	14	185	13.21	13.21	7	13	2.333
Funder, John W.	Hudson Institute of Medical Research	Australia	13	12	1535	118.08	127.92	12	13	0.353
Lu, Yanjie	Harbin Medical University	China	12	11	1097	91.42	99.73	10	12	0.714
Young, Morag J.	Hudson Institute of Medical Research	Australia	12	12	1506	125.50	125.50	12	12	0.375
Tu, Bin	Anhui Medical University	China	12	12	169	14.08	14.08	6	12	1.500
Song, Kai	Anhui Medical University	China	12	12	169	14.08	14.08	6	12	1.500
Liu, Peiqing	Jinan University	China	11	10	288	26.18	28.80	8	11	0.500
Ge, Junbo	Fudan University	China	11	11	267	24.27	24.27	8	11	0.571
Li, Jun	Anhui Medical University	China	10	9	363	36.30	40.33	8	10	0.615
Zhang, Youyi	Peking University	China	10	10	517	51.70	51.70	8	10	0.500
Shan, Hongli	Harbin Medical University	China	9	9	564	62.67	62.67	7	9	0.500
Ding, Xuan Sheng	China Pharmaceutical University	China	9	8	213	23.67	26.63	8	9	1.000
Li, Huihua	Capital Medical University	China	9	9	929	103.22	103.22	9	9	0.600
Zhang, Ye	Anhui Medical University	China	9	9	149	16.56	16.56	5	9	1.250
Zhou, Yang	Anhui Medical University	China	9	9	142	15.78	15.78	5	9	1.250
Du, Jie	Capital Medical University	China	9	9	929	103.22	103.22	9	9	0.600
Huang, Chih Yang	China Medical University	Taiwan	9	8	150	16.67	18.75	8	9	0.381
Yang, Baofeng	Harbin Medical University	China	9	9	875	97.22	97.22	7	9	0.368
Ma, Zhen Guo	Wuhan University	China	9	9	849	94.33	94.33	9	9	0.818

C/CP = average citations per cited publication, C/P = average citations per publication, *g* = *g*-index, *h* = *h*-index, *m* = *m*-index, NCP = number of cited publications, TC = total citations, TP = total number of publications.

Source: Generated by the author(s) using biblioMagika (Ahmi^[15]^).

Institutions in China produce the overwhelming majority of the most productive authors, headed by Anhui Medical University. In particular, the list includes Tao Hui, Yang Jing Jing, Liu Zhi Yan, Lin Li Chan, Tu Bin, and Song Kai of Anhui Medical University, as well as Li Jun, Zhou Yang, and Zhang Ye of Anhui Medical University, showing that this university is a local epicenter of research into cardiac fibrosis. The tendency of clustering of authors is an indicator of the presence of a research group or center of excellence focused on a particular topic and potentially benefiting from sustained institutional or national funding systems. Additional leading Chinese institutions with a strong author representation are Wuhan University (e.g., Tang Qi Zhu, Ma Zhen Guo), Harbin Medical University (e.g., Lu Yanjie, Yang Baofeng, Shan Hongli), Capital Medical University (e.g., Du Jie, Li Huihua), Nanjing University of Chinese Medicine, Peking University, and Fudan University.

While most productive authors are from China, 2 notable authors from Australia, John W. Funder and Morag J. Young, both affiliated with the Hudson Institute of Medical Research, stand out due to their exceptionally high citation impact. Despite having only 13 and 12 publications, respectively, they have accumulated 1535 and 1506 citations, yielding average citations per publication (C/P) of 118.08 and 125.50, far surpassing their peers. This suggests that their work is highly regarded and likely foundational or influential within the field.

The international contribution was also noticed from Taiwan. Huang Chih Yang, from China Medical University, appears among the top contributors. This further reinforces the regional distribution of influential authors within the broader Asia–Oceania framework. While total publications provide a measure of productivity, the C/P, average citations per cited publication (C/CP), and *h*-, *g*-, and *m*-indices offer insights into scholarly impact and consistency. First, Tang Qi Zhu (Wuhan University) stands out with a high C/P of 48.77, total citations (TC) of 1073, and a solid *h*-index of 14, indicating both prolific output and substantial influence. Second, Li Huihua and Du Jie (Capital Medical University) each have only 9 publications, yet have gathered 929 citations, demonstrating exceptionally high-impact publications (C/P = 103.22). Third, Lu Yanjie and Ma Zhen Guo also exhibit high citation averages (above 90), reinforcing the qualitative significance of their work despite fewer publications.

On the other hand, the *m*-index, which normalizes the *h*-index by the number of years since the first publication, reflects sustained scholarly output. By referring to Liu Zhi Yan and Lin Li Chan (both with *m* = 2.333), they exhibit the highest *m*-indices among the group. This indicates that their impact has been achieved within a relatively short publication window, suggesting rising prominence. Authors like Zhao Jian Yuan and Tu Bin have relatively high *m*-indices (1.5–1.6), implying an ongoing upward trajectory in their academic influence. In contrast, authors with very high C/P but low *m*-index (e.g., Funder, Young) may have published earlier seminal works that remain highly cited but are less active in recent years.

Table 7 concludes a small but influential group of authors who are shaping the field of cardiac fibrosis research in Asia and Oceania. Chinese authors, particularly those from Anhui Medical University, dominate in terms of sheer publication volume, whereas Australian researchers contribute disproportionately in terms of citation impact. The combined analysis of productivity and influence suggests a mature and growing scholarly ecosystem, with several rising stars and a few well-established authorities whose work forms the intellectual foundation of the field. The inclusion of multiple institutions and countries indicates an increasingly collaborative and geographically diversified research landscape.

### 4.4. Publication by institutions

Table 8 presents a bibliometric profile of the most productive institutions contributing to cardiac fibrosis research, each with a minimum of 5 publications. The table includes metrics that reflect not only the TP but also the impact and consistency of scholarly output, such as TC, average C/P, *h*-index, *g*-index, and *m*-index. The institutions are primarily located in China, with a few prominent entries from Australia, Taiwan, and Japan.

**Table 8:** Most productive institutions with a minimum of 5 publications.

Affiliation	Country	TP	NCA	NCP	TC	C/P	C/CP	*h*	*g*	*m*
Harbin Medical University	China	56	618	54	2312	41.29	42.81	25	48	1.316
Shanghai Jiao Tong University	China	55	260	54	1267	23.04	23.46	19	35	1.357
Nanjing Medical University	China	53	233	49	1291	24.36	26.35	20	35	1.818
Anhui Medical University	China	46	261	43	1162	25.26	27.02	20	34	1.538
Wuhan University	China	45	265	41	1421	31.58	34.66	20	37	1.176
Fudan University	China	38	200	35	936	24.63	26.74	17	30	0.708
Shandong University	China	35	271	30	1005	28.71	33.50	16	31	1.000
Sun Yat-sen University	China	35	227	30	857	24.49	28.57	17	29	1.063
Capital Medical University	China	31	150	30	1494	48.19	49.80	19	31	1.056
Monash University	Australia	30	97	30	1768	58.93	58.93	19	30	0.826
Huazhong University of Science and Technology	China	28	160	25	625	22.32	25.00	13	25	0.765
Peking University	China	25	161	25	1083	43.32	43.32	17	25	0.895
Zhejiang University	China	24	139	21	672	28.00	32.00	12	24	0.857
Baker Heart and Diabetes Institute	Australia	23	59	22	1837	79.87	83.50	17	23	0.500
Southern Medical University	China	21	86	18	380	18.10	21.11	10	19	0.455
China Pharmaceutical University	China	20	66	18	453	22.65	25.17	13	20	1.444
China Medical University	Taiwan	18	53	17	897	49.83	52.76	13	18	0.619
Zhengzhou University	China	18	120	17	538	29.89	31.65	12	18	1.000
Central South University	China	17	78	16	524	30.82	32.75	11	17	0.524
Jilin University	China	16	69	15	285	17.81	19.00	10	16	0.714
Tongji University	China	16	60	15	314	19.63	20.93	9	16	0.750
University of Melbourne	Australia	15	54	14	891	59.40	63.64	13	15	0.565
Xi’an Jiaotong University	China	14	70	14	329	23.50	23.50	10	14	0.909
Osaka University	Japan	14	111	14	766	54.71	54.71	12	14	0.522
Nanjing University of Chinese Medicine	China	14	59	14	438	31.29	31.29	11	14	1.100

C/CP = average citations per cited publication, C/P = average citations per publication, *g* = *g*-index, *h* = *h*-index, *m* = *m*-index, NCA = number of contributing authors, NCP = number of cited publications, TC = total citations, TP = total number of publications.

Source: Generated by the author(s) using biblioMagika (Ahmi^[15]^).

The first aspect is the leading institutions by publication volume and impact. Harbin Medical University (China) leads in total publications (TP = 56), with high citation impact (TC = 2312) and a strong *h*-index (25) and *g*-index (48). Its high C/P (41.29) and *m*-index (1.316) suggest sustained productivity and influence over time. Next, Shanghai Jiao Tong University (TP = 55) follows closely in publication count. Despite a slightly lower citation impact (TC = 1267; C/P = 23.04), it maintains a robust scholarly profile with an *h*-index of 19 and a strong *m*-index (1.357), indicating steady long-term productivity. For Nanjing Medical University (TP = 53), it demonstrates a balanced profile with relatively high total citations (TC = 1291) and an impressive *m*-index (1.818), one of the highest in the table, suggesting a highly consistent and impactful research output per year.

The second aspect is the research powerhouses with high-impact per paper. Baker Heart and Diabetes Institute (Australia) stands out for its exceptional citation impact, despite fewer publications (TP = 23). It has the highest C/P (79.87) and C/CP (83.5), alongside a strong *h*-index (17), indicating that its research is highly influential, likely producing foundational or groundbreaking studies. University of Melbourne (TP = 15) and Osaka University (TP = 14) also exhibit strong C/P values (59.40 and 54.71, respectively), reflecting the high scholarly value of fewer but well-cited publications. Besides, Capital Medical University (China) also shows a significant impact with a high C/P (48.19) and C/CP (49.80), reflecting quality over quantity.

The third aspect is the dominance of Chinese institutions. Of the 25 listed institutions, 21 are from China, highlighting China’s central role in advancing cardiac fibrosis research. Institutions such as Anhui Medical University, Wuhan University, Fudan University, and Shandong University are significant contributors in both quantity and citation impact. Notably, Anhui Medical University (TP = 46) maintains high productivity with a moderate-to-high impact (TC = 1162; *h* = 20; *m* = 1.538), reinforcing its status as a key institutional player, especially given its presence in the most productive authorship list (Table 7).

The fourth aspect, notable international contributors. Monash University (TP = 30; C/P = 58.93) from Australia is notable for combining high impact with moderate output, suggesting focused excellence in cardiac fibrosis research. China Medical University in Taiwan (TP = 18; C/P = 49.83) shows strong performance, reflecting the contribution of regional institutions outside mainland China. Meanwhile, Osaka University (Japan) maintains a high-impact profile, although with fewer publications, underscoring Japan’s strategic contribution to the field.

The *h*-index and *g*-index reflect both the volume and consistency of high-impact publications. Institutions such as Harbin Medical University (*h* = 25, *g* = 48) and Wuhan University (*h* = 20, *g* = 37) have high values, demonstrating broad influence in the literature. In the perspective of the *m*-index, which normalizes productivity by career length, it reveals institutions with sustained annual productivity. Nanjing Medical University *(m* = 1.818), China Pharmaceutical University (*m* = 1.444), and Anhui Medical University (*m* = 1.538) rank highly, suggesting strong and consistent performance over time, rather than a few recent spikes in output.

In summary, Table 8 illustrates that cardiac fibrosis research is dominated by Chinese institutions, which demonstrate not only productivity but also substantial impact, with a few select institutions in Australia, Taiwan, and Japan contributing highly cited, high-quality research. Institutions such as Harbin Medical University, Nanjing Medical University, and Baker Heart and Diabetes Institute emerge as particularly influential due to their combination of high publication volume, citation impact, and index performance. The variation in citation metrics and indexes also highlights differing research strategies, some prioritize volume, while others focus on impactful and high-quality studies.

### 4.5. Publication by countries

Table 9 provides a comprehensive bibliometric overview of countries contributing to cardiac fibrosis research. It evaluates not only the TP but also the impact (citations), productivity indices (*h*-, *g*-, *m*-indices), and citation quality metrics (C/P, C/CP). The analysis reveals geographic patterns, research leadership, and variability in influence and efficiency of publication outputs.

**Table 9:** The top countries contributed to the publications.

Country	TP	NCA	NCP	TC	C/P	C/CP	*h*	*g*	*m*
China	749	6000	687	19,772	26.40	28.78	68	140	2.833
Japan	88	670	84	4160	47.27	49.52	35	64	0.854
Australia	59	289	57	3661	62.05	64.23	33	59	0.971
Taiwan	35	264	34	1448	41.37	42.59	19	35	0.905
India	17	79	16	236	13.88	14.75	9	15	0.321
South Korea	13	134	11	465	35.77	42.27	11	13	0.688
Iran	12	55	7	112	9.33	16.00	5	10	0.556
Indonesia	8	37	4	14	1.75	3.50	3	3	0.500
Turkey	7	40	4	27	3.86	6.75	4	5	0.235
Country NA	6	15	6	335	55.83	55.83	5	6	0.357
Hong Kong	6	17	5	427	71.17	85.40	5	6	0.357
Singapore	4	9	4	55	13.75	13.75	3	4	0.333
Thailand	4	6	4	219	54.75	54.75	4	4	0.400
Israel	3	11	3	34	11.33	11.33	3	3	0.600
Malaysia	2	5	2	77	38.50	38.50	2	2	0.167
Lebanon	2	6	2	31	15.50	15.50	2	2	0.333
New Zealand	2	2	2	7	3.50	3.50	2	2	0.125
Nepal	1	1	1	16	16.00	16.00	1	1	0.250
Kazakhstan	1	5	1	30	30.00	30.00	1	1	0.250
Macao	1	3	1	10	10.00	10.00	1	1	0.250
Oman	1	4	0	0	0.00	0.00	0	0	0.000

C/CP = average citations per cited publication, C/P = average citations per publication, *g* = *g*-index, *h* = *h*-index, *m* = *m*-index, NCA = number of contributing authors, NCP = number of cited publications, TC = total citations, TP = total number of publications.

Source: Generated by the author(s) using biblioMagika (Ahmi^[15]^).

China is the undisputed leader, contributing 749 publications and accounting for the vast majority of global output in this field. It also leads in total citations (TC = 19,772) and maintains strong performance across bibliometric indices (*h* = 68, *g* = 140, *m* = 2.833). While its average citations per publication (C/P = 26.40) is lower than that of some countries with fewer publications, this is expected for large-volume contributors. The high *h*-index (68) and *g*-index (140) affirm China’s both broad and deep impact, and its *m*-index (2.833) underscores consistent productivity over time.

Several countries exhibit fewer publications but higher citation efficiency, indicating a focus on high-quality or groundbreaking research. First, Australia (TP = 59) has the highest citation impact (C/P = 62.05; C/CP = 64.23), total citations = 3661, and robust indices (*h* = 33; *m* = 0.971), suggesting that its research is consistently high in quality and influence. Second, Japan (TP = 88) has strong citation performance (C/P = 47.27; TC = 4160), with a high *h*-index (35) and *g*-index (64), indicating both productivity and a stable body of influential research. Its *m*-index (0.854) is moderate, suggesting steady but not accelerating annual impact. Third, Taiwan (TP = 35; C/P = 41.37) also demonstrates strong citation metrics relative to its output, with a solid *h*-index (19) and *m*-index (0.905), indicating efficient and impactful research activity.

There is an emerging contributor in Asia and Southwest Asia. First, South Korea (TP = 13) shows notable efficiency (C/P = 35.77), with good citation per cited publication (C/CP = 42.27), reflecting that its few publications are well-received. Second, India (TP = 17) is an emerging contributor with moderate productivity and a relatively lower citation impact (C/P = 13.88). Its indices (*h* = 9, *m* = 0.321) suggest early-stage or developing engagement in this research field. Meanwhile, Iran, Indonesia, Turkey, and Malaysia show low publication and citation counts, indicating nascent or sporadic involvement in cardiac fibrosis research. For instance, Iran’s C/P = 9.33, and Turkey C/P = 3.86 suggest that further development in research infrastructure or collaboration may be needed.

Nevertheless, smaller countries with noteworthy citation performance have depicted Hong Kong (TP = 6; C/P = 71.17) and Thailand (TP = 4; C/P = 54.75) as exhibiting very high citation efficiency, implying focused and impactful contributions, possibly through collaborations or publication in high-impact journals. Country NA (unspecified) shows a high citation average (C/P = 55.83), suggesting possibly highly cited multinational collaborations or data errors in country attribution.

There is observable citation performance across other territories. Countries such as Singapore, Israel, Lebanon, and New Zealand contribute minimally (TP = 2–4), with varied citation performance. While some (e.g., Israel, C/P = 11.33) suggest potential for growth, others (e.g., New Zealand, C/P = 3.50) appear marginal. Several countries (e.g., Oman, with TP = 1 and 0 citations) reflect incipient engagement or isolated studies with negligible impact thus far. In terms of analysis of indices, China maintains the highest values for all 3 indices, particularly the *m*-index (2.833), suggesting sustained and increasing annual scholarly impact. Japan, Australia, and Taiwan also report balanced *h*- and *g*-index values, reinforcing their reputations for research stability and influence, even if publication counts are lower. For smaller countries or those with recent entry into the field, *m*-index values are typically lower (e.g., Nepal, Kazakhstan, Macao at 0.250), reflecting their shorter timeframes or limited cumulative influence.

Overall, Table 9 illustrates that while China dominates cardiac fibrosis research in terms of volume, countries such as Australia, Japan, Taiwan, and Hong Kong lead in research impact per publication. This reflects the diversity of contributions: high-volume producers, high-impact specialists, and emerging contributors. The global research landscape is thus marked by regional leadership in East Asia and Oceania, with potential for expansion and collaboration across South and Southeast Asia, the Middle East, and beyond. Strategic international partnerships could amplify both research output and quality, especially for countries with promising citation efficiency but lower publication counts.

**Figure 4.**
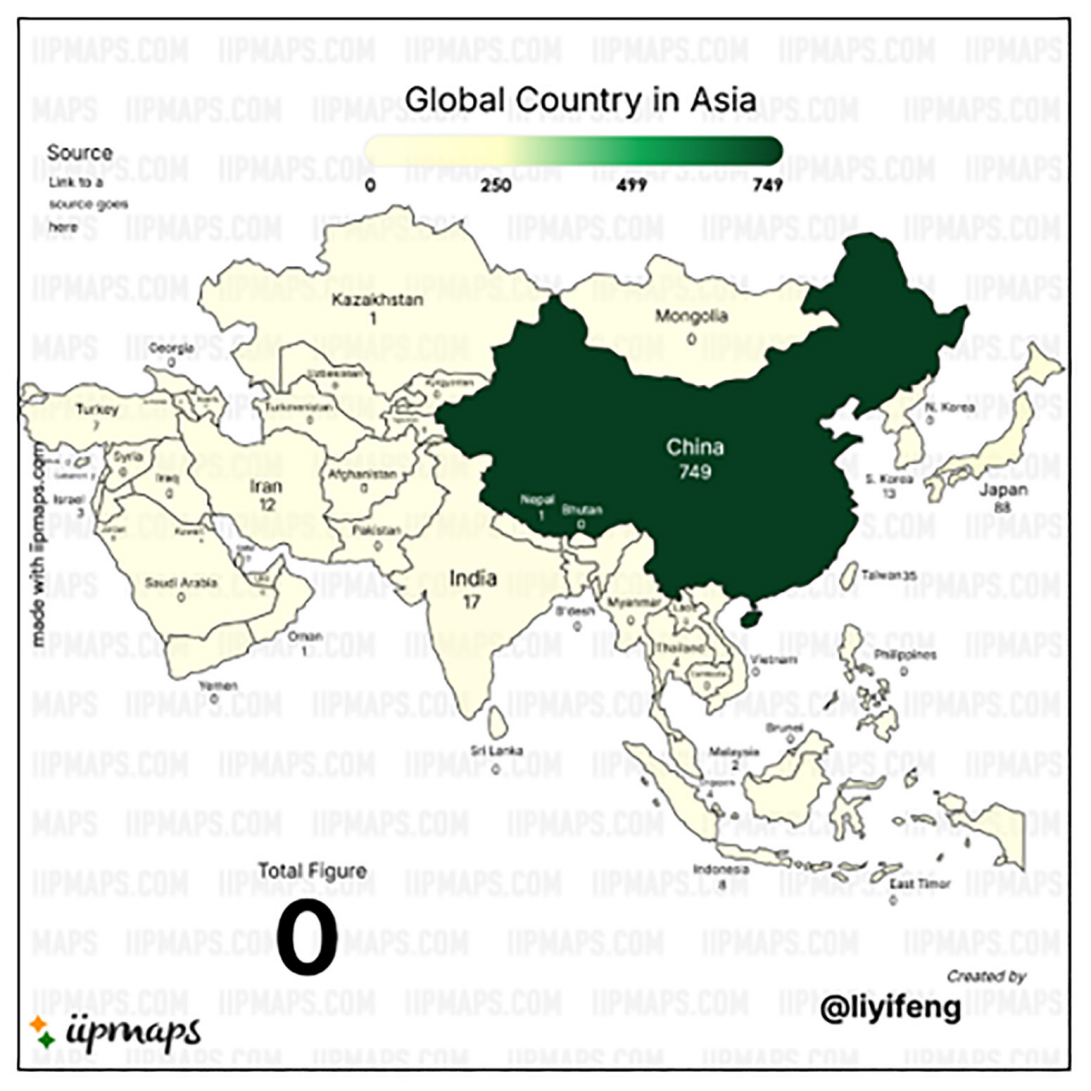
Asia’s scientific production indexed by Scopus on cardiac fibrosis. This map visualizes the number of publications from Asian countries indexed in Scopus on cardiac fibrosis between 1985 and 2025. China is the leading contributor with 749 publications. The color gradient reflects publication volume. Source: Generated by the author(s) using iipmaps.com.

**Figure 5.**
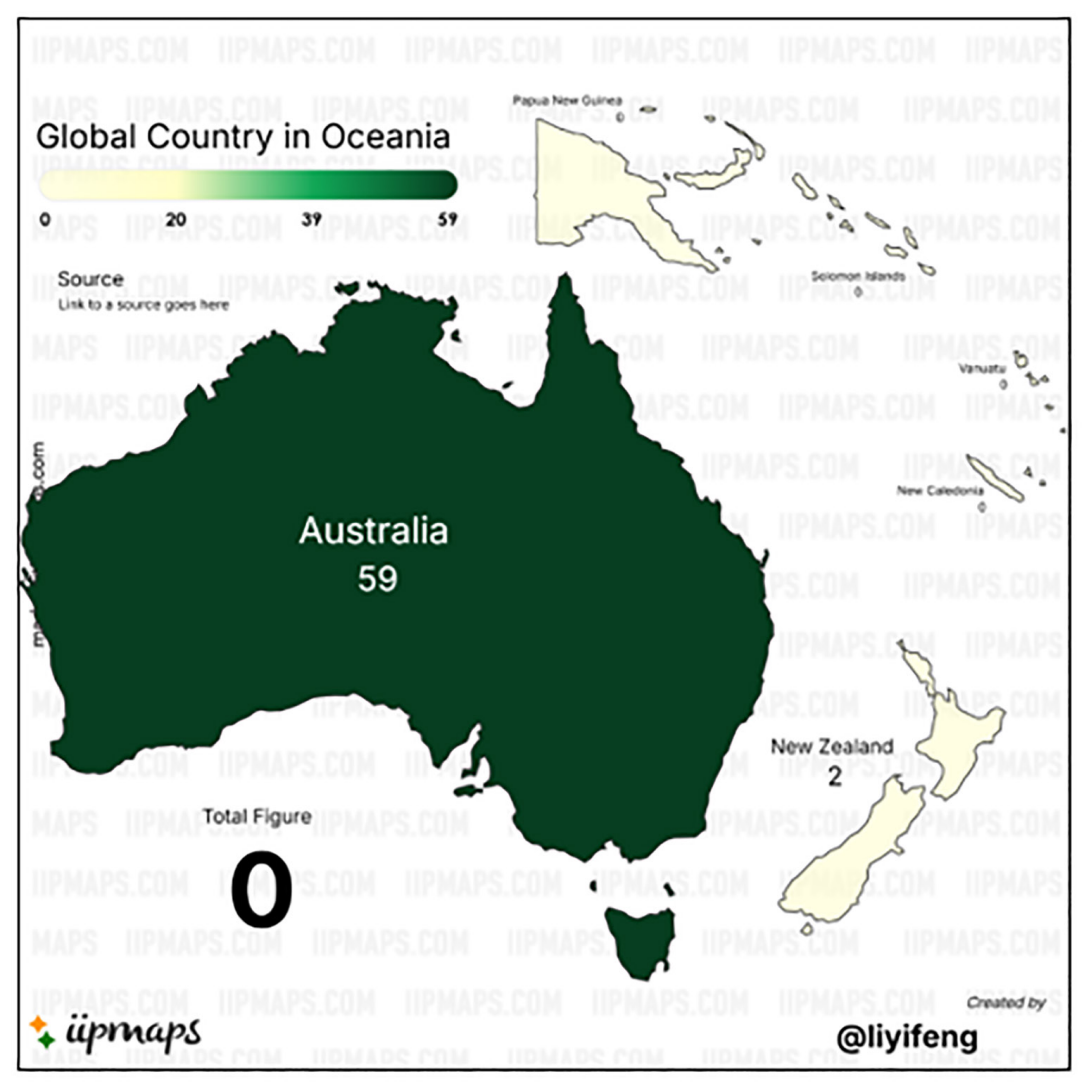
Oceania’s scientific production indexed by Scopus on cardiac fibrosis. This map shows the number of publications on cardiac fibrosis from Oceania countries indexed in Scopus between 1985 and 2025. Australia leads the region with 59 publications. The color gradient reflects publication volume. Source: Generated by the author(s) using iipmaps.com.

Figures 4 and 5 offer a visual representation of country-level contributions in the Asia and Oceania region to the field of cardiac fibrosis, consistent with the bibliometric statistics detailed in Table 9. The figures use color intensity and/or node size to reflect the volume of publications from each country, likely incorporating citation strength or co-authorship links in the visualization algorithm. China is unmistakably the central node in the Asia continent (Fig. 4), while Australia is the prominent central node in Oceania (Fig. 5).

### 4.6. Publication by titles

Table 10 presents a detailed bibliometric analysis of the most active journals (i.e., source titles) that have published 20 or more documents related to cardiac fibrosis. The data provide a multi-dimensional perspective on source productivity, influence, and temporal research intensity.

**Table 10:** Most active source titles that published 20 or more documents.

Source title	TP	NCA	NCP	TC	C/P	C/CP	*h*	*g*	*m*
Frontiers in Pharmacology	20	20	20	542	27.10	27.10	15	20	1.667
European Journal of Pharmacology	19	19	19	302	15.89	15.89	10	17	0.909
International Journal of Cardiology	17	17	17	377	22.18	22.18	10	17	0.769
Biochemical and Biophysical Research Communications	17	17	15	323	19.00	21.53	13	17	1.182
PLoS ONE	17	17	15	1032	60.71	68.80	13	17	0.929
International Journal of Molecular Sciences	16	16	14	548	34.25	39.14	11	16	1.222
Biomedicine and Pharmacotherapy	15	15	15	480	32.00	32.00	12	15	0.706
International Immunopharmacology	14	14	9	181	12.93	20.11	5	13	0.625
Frontiers in Cardiovascular Medicine	14	14	13	399	28.50	30.69	9	14	1.000
Life Sciences	12	12	10	288	24.00	28.80	7	12	0.318
Acta Pharmacologica Sinica	12	12	12	383	31.92	31.92	9	12	0.474
Scientific Reports	11	11	11	482	43.82	43.82	9	11	0.818
Theranostics	11	11	11	400	36.36	36.36	9	11	1.000
Oxidative Medicine and Cellular Longevity	10	10	10	207	20.70	20.70	8	10	0.667
Cellular Physiology and Biochemistry	10	10	10	686	68.60	68.60	10	10	0.769
Evidence-based Complementary and Alternative Medicine	10	10	10	146	14.60	14.60	8	10	1.000
Phytomedicine	10	10	10	267	26.70	26.70	8	10	0.500
Journal of Cardiovascular Pharmacology	9	9	9	107	11.89	11.89	5	9	0.208
Journal of Cellular and Molecular Medicine	9	9	9	591	65.67	65.67	7	9	0.500
Molecular Medicine Reports	9	9	9	220	24.44	24.44	8	9	0.571

C/CP = average citations per cited publication, C/P = average citations per publication, *g* = *g*-index, *h* = *h*-index, *m* = *m*-index, NCA = number of contributing authors, NCP = number of cited publications, TC = total citations, TP = total number of publications.

Source: Generated by the author(s) using biblioMagika (Ahmi^[15]^).

The journal with the highest number of publications is Frontiers in Pharmacology, with a number of cited publications of 20. It has also amassed a respectable 542 citations, averaging 27.10 citations per paper, and achieved a high *h*-index of 15. The *m*-index of 1.667, which adjusts the *h*-index for career length, suggests a sustained and recent scholarly contribution, reinforcing its central role in disseminating cardiac fibrosis research. Other top contributors include European Journal of Pharmacology (TP = 19; C/P = 15.89), International Journal of Cardiology (TP = 17; C/P = 22.18), and Biochemical and Biophysical Research Communications and PLoS ONE (each TP = 17). These journals represent established biomedical platforms, particularly in pharmacology, cardiology, and general biological sciences.

While productivity indicates breadth, citation metrics offer insight into scholarly influence, that is, PLoS ONE stands out with an exceptional 1032 total citations across 17 papers, yielding the highest average citations per publication (C/P = 60.71) and per cited paper (C/CP = 68.80). This suggests its publications on cardiac fibrosis are highly impactful, despite the journal’s broad scope and multidisciplinary nature. Cellular Physiology and Biochemistry and Journal of Cellular and Molecular Medicine also exhibit notable citation density, with C/P values of 68.60 and 65.67, respectively. These results imply that although their volume of output is modest (TP = 10 and 9), their influence per article is disproportionately high, indicating selectivity and relevance in the field. Similarly, Scientific Reports, Theranostics, and International Journal of Molecular Sciences also record C/P values exceeding 30, underscoring their role in publishing high-impact studies.

The *h*-, *g*-, and *m*-indices offer further insight into the sustained scholarly performance of each journal. Frontiers in Pharmacology holds the highest *h*-index (*h* = 15), indicating that 15 of its publications on cardiac fibrosis have been cited at least 15 times, evidence of consistent influence across a breadth of articles. Several journals exhibit high *g*-indices (e.g., 20 for Frontiers in Pharmacology, 17 for European Journal of Pharmacology and International Journal of Cardiology), indicating a concentration of citation impact among the most cited articles. The *m*-index, which accounts for the recency of impact, is highest for Frontiers in Pharmacology (1.667), followed by International Journal of Molecular Sciences (1.222) and Biochemical and Biophysical Research Communications (1.182), indicating these journals have recently emerged as hubs of activity in cardiac fibrosis research.

The data suggest that both specialized and broad-scope journals play important roles. Subject-specific journals such as Frontiers in Cardiovascular Medicine, Journal of Cardiovascular Pharmacology, and Acta Pharmacologica Sinica serve as dedicated outlets for focused cardiac fibrosis content. Conversely, the visibility and citation potential are wide-reaching in multidisciplinary journals, such as PLoS ONE, Scientific Reports, and International Journal of Molecular Sciences, which may draw a cross-disciplinary readership.

This analysis documented that cardiac fibrosis research is published in a wide range of journals, including high-volume journals and high-impact journals. The examples of such journals include Frontiers in Pharmacology, PLoS ONE, and International Journal of Molecular Sciences, characterized by the balance of output and citation depth. In the meantime, the journals that have fewer published articles but with a high average citation, such as Cellular Physiology and Biochemistry, emphasize the qualitative distinction of certain publications. Beyond this, the predominance of journals with pharmacologic, cardiologic/physiologic, molecular, and biomedical bases is characteristic of the inherently interdisciplinary nature of cardiac fibrosis research that cuts across clinical perspectives and research of mechanistic origin. This finding is fundamental both to researchers seeking to determine the best outlets to publish and to know how knowledge in the field is publicized and rewarded in the international scientific world.

### 4.7. Highly cited documents

Table 11 presents the 20 most highly cited articles in the domain of cardiac fibrosis, highlighting their bibliographic details (authors, title, source journal), total citations, and citations per year.^[18–37]^ This data is essential in identifying landmark studies, key contributors, and thematic trends that have

**Table 11:** Top 20 highly cited articles.

No.	Authors	Title	Source title	Cites	Cites per year
1	Tamura et al^[18]^	Cardiac fibrosis in mice lacking brain natriuretic peptide	Proceedings of the National Academy of Sciences of the United States of America	541	1
2	Young et al^[19]^	Mineralocorticoids, hypertension, and cardiac fibrosis	Journal of Clinical Investigation	440	2
3	Lee et al^[20]^	Dapagliflozin, a selective SGLT2 Inhibitor, attenuated cardiac fibrosis by regulating the macrophage polarization via STAT3 signaling in infarcted rat hearts	Free Radical Biology and Medicine	427	3
4	Pan et al^[21]^	MicroRNA-101 inhibited post-infarct cardiac fibrosis and improved left ventricular compliance via the FBJ osteosarcoma oncogene/transforming factor-β1 pathway	Circulation	289	4
5	Ma et al^[22]^	Cardiac fibrosis: New insights into the pathogenesis	International Journal of Biological Sciences	278	5
6	Zhang et al^[23]^	miR-29b as a therapeutic agent for angiotensin II-induced cardiac fibrosis by targeting TGF-β/Smad3 signaling	Molecular Therapy	267	6
7	Rickard et al^[24]^	Deletion of mineralocorticoid receptors from macrophages protects against deoxycorticosterone/salt-induced cardiac fibrosis and increased blood pressure	Hypertension	259	7
8	Wang et al^[25]^	MicroRNA-24 regulates cardiac fibrosis after myocardial infarction	Journal of Cellular and Molecular Medicine	253	8
9	Ma et al^[26]^	Macrophage-stimulated cardiac fibroblast production of IL-6 is essential for TGF-β/Smad activation and cardiac fibrosis induced by angiotensin II	PLoS ONE	252	9
10	Young et al^[27]^	Determinants of cardiac fibrosis in experimental hypermineralocorticoid states	American Journal of Physiology – Endocrinology and Metabolism	250	10
11	Xiao et al^[28]^	Metformin attenuates cardiac fibrosis by inhibiting the TGF-β-Smad3 signaling pathway	Cardiovascular Research	211	11
12	Li et al^[29]^	Polystyrene microplastics cause cardiac fibrosis by activating Wnt/β -catenin signaling pathway and promoting cardiomyocyte apoptosis in rats	Environmental Pollution	211	12
13	Yue et al^[30]^	Transforming growth factor beta (TGF-β) mediates cardiac fibrosis and induces diabetic cardiomyopathy	Diabetes Research and Clinical Practice	207	13
14	Tzanidis et al^[31]^	Direct actions of urotensin II on the heart: Implications for cardiac fibrosis and hypertrophy	Circulation Research	203	14
15	Qu et al^[32]^	MIAT is a pro-fibrotic long noncoding RNA governing cardiac fibrosis in post-infarct myocardium	Scientific Reports	196	15
16	Matsumoto et al^[33]^	Chymase inhibition prevents cardiac fibrosis and improves diastolic dysfunction in the progression of heart failure	Circulation	195	16
17	Yuan et al^[34]^	Mir-21 promotes cardiac fibrosis after myocardial infarction via targeting Smad7	Cellular Physiology and Biochemistry	192	17
18	Tomita et al^[35]^	Early induction of transforming growth factor-β via angiotensin II type 1 receptors contributes to cardiac fibrosis induced by long-term blockade of nitric oxide synthesis in rats	Hypertension	182	18
19	Fujita et al^[36]^	Adiponectin protects against angiotensin II-induced cardiac fibrosis through activation of PPAR-α	Arteriosclerosis, Thrombosis, and Vascular Biology	178	19
20	Qin et al^[37]^	Role of PI3K/Akt signaling pathway in cardiac fibrosis	Molecular and Cellular Biochemistry	176	20

AKT = protein kinase B, FBJ = osteosarcoma oncogene (c-Fos), IL-6 = interleukin-6, PI3K = phosphoinositide 3-kinase, PPAR-α = peroxisome proliferator-activated receptor alpha, SGLT2 = sodium–glucose cotransporter 2, STAT3 = signal transducer and activator of transcription 3, TGF-β = transforming growth factor beta.

Source: Generated by the author(s) using biblioMagika (Ahmi^[15]^).

The top-cited article is Tamura et al,^[18]^ titled “Cardiac fibrosis in mice lacking brain natriuretic peptide,” published in Proceedings of the National Academy of Sciences of the United States of America, with 541 citations. This foundational study explored the genetic underpinnings of cardiac fibrosis, specifically the role of natriuretic peptides in modulating fibrotic response, and remains a pivotal reference in the field. Other highly influential studies include Young et al^[19]^ with 440 citations, investigating the impact of mineralocorticoids and hypertension on cardiac fibrosis (Journal of Clinical Investigation) and Lee et al^[20]^ with 427 citations, reporting that dapagliflozin, a sodium–glucose cotransporter 2 inhibitor, attenuates cardiac fibrosis via signal transducer and activator of transcription 3-mediated macrophage polarization (Free Radical Biology and Medicine). These papers collectively underscore a strong emphasis on molecular pathways, pharmacological modulation, and animal models of cardiac fibrosis.

The list reveals key molecular mechanisms and therapeutic targets frequently associated with high-impact studies. Firstly, TGF-β/Smad signaling pathway. Appears in multiple top-cited articles^[21,23,26,28,30]^ confirming its central role in fibrogenesis and its relevance in both mechanistic and translational research. Secondly, microRNAs and long noncoding RNAs (lncRNAs). Several articles report their regulatory functions in post-infarction fibrosis, including *miR-101*,^[21]^ *miR-29b*,^[23]^ *miR-24*,^[25]^ *miR-21*^,[34]^ and *MIAT lncRNA*.^[32]^ This highlights a growing interest in epigenetic and post-transcriptional regulation in fibrosis. In addition, pharmacological interventions are also noted in articles on dapagliflozin, metformin, chymase inhibition, peroxisome proliferator-activated receptor alpha activation, and adiponectin, reflecting ongoing exploration into cardioprotective agents with anti-fibrotic potential.

While total citations gauge historical impact, citation rate (citations/year) is a useful proxy for current relevance and emerging influence. Lee et al^[20]^ recorded ~71 citations/year (427 in 6 years), Ma et al^[22]^ charted 278 citations in ~5 years, and Qin et al^[37]^ reported 176 citations in just 2 to 3 years (~88/year), suggesting strong ongoing interest. These high citation rates reflect that recent publications especially those addressing molecular mechanisms or novel therapeutic agents are rapidly gaining influence, potentially shaping future research and clinical strategies.

These highly cited studies are published in a range of high-impact journals across multiple disciplines. First, cardiology and vascular biology field in Circulation, Circulation Research, Hypertension, International Journal of Cardiology. Second, molecular and cellular biology in Molecular Therapy, Journal of Cellular and Molecular Medicine, Cellular Physiology and Biochemistry. Lastly, multidisciplinary science in PNAS, Scientific Reports, PLoS ONE. This dispersion suggests that cardiac fibrosis is an interdisciplinary research topic, attracting attention from both clinical and basic science communities.

In summary, these findings underscore the scientific and clinical significance of cardiac fibrosis and delineate the core knowledge base upon which current research builds. The increasing citation rates of more recent studies suggest a dynamic and rapidly evolving research landscape, marked by advances in molecular biology, pharmacotherapy, and systems medicine.

### 4.8. Co-authorship analysis

**Figure 6.**
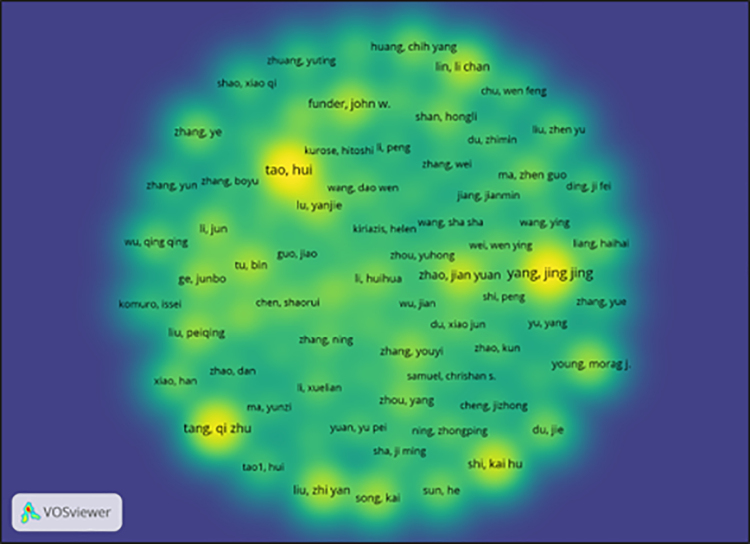
Density visualization of the author’s co-authorship analysis. This figure displays the density map of co-authorship networks among authors publishing on cardiac fibrosis in Asia and Oceania. Brighter areas indicate clusters with stronger collaboration activity. Visualization was generated using VOSviewer. Source: Generated by the author(s) using VOSviewer (Van Eck & Waltman^[16]^).

In bibliometric research, co-authorship analysis identifies and visualizes collaborative relationships among authors based on their joint contributions to academic publications. In this context, VOSviewer constructs a network where nodes represent individual authors, and links denote coauthored documents (Fig. 6). This form of analysis is instrumental in uncovering the collaborative structure, identifying prolific scholars, and detecting intellectual communities or research clusters within a specific field.

For this analysis, the full counting method was applied in VOSviewer. Under this method, each coauthored paper is counted equally for all contributing authors, regardless of their order of appearance. To ensure meaningful interpretation and to reduce visual clutter, a minimum threshold of 5 documents per author was established. Out of an initial set of 5991 authors, only 78 authors met this threshold and were thus included in the final visualization. This selection criterion enables a focused examination of the most active contributors within the domain. The link to the VOSviewer file for the co-authorship density map is provided in Appendix B.

Figure 6 employs a density visualization format, wherein the color intensity reflects the local concentration of authors and their co-authorship interactions. Yellow zones indicate regions with a high density of publications and collaborations, representing clusters of influential and highly interconnected scholars. Green and blue zones correspond to areas with lower collaboration density, often signifying peripheral or less active contributors. The spatial proximity between authors signifies the strength of their collaborative ties; authors located close to one another are more likely to have coauthored multiple papers or belong to the same intellectual network.

Tao, Hui, Tang, Qi Zhu, Yang, Jing Jing, and Zhao, Jian Yuan are at the center of bright yellow regions, signifying their prominence in the field through both high publication volume and frequent collaboration. For instance, Tao, Hui has authored 30 documents and received 28 citations, while Tang, Qi Zhu has 22 documents, making them central figures in the network. Zhao, Jian Yuan (15 publications, 132 citations) shows a powerful scholarly impact, as evidenced by both high productivity and citation count.

Authors appear grouped into distinct clusters, indicating specialized research teams or thematic affiliations. While the clusters are not explicitly color-coded in the density map, their spatial grouping reflects thematic or institutional collaborations. For peripheral nodes (in cooler colors such as green and blue), include authors like Young, Morag J., Chu, Wen Feng, and Ma, Zhen Guo, who, despite possibly high individual impact (e.g., Young, Morag J. with 287 citations), may have fewer co-authorship connections within this specific network.

Authors such as Li, Huihua, Wang, Dao Wen, and Lu, Yanjie are positioned near central figures, suggesting secondary roles in collaborative groups or bridging positions across multiple clusters. The visualization also identifies less-integrated researchers, indicated by authors situated on the outer rim with fewer visible linkages, such as Wu, Qing Qing, or Xiao, Han, suggesting isolated or emerging research contributions.

### 4.9. Co-occurrence analysis

**Figure 7.**
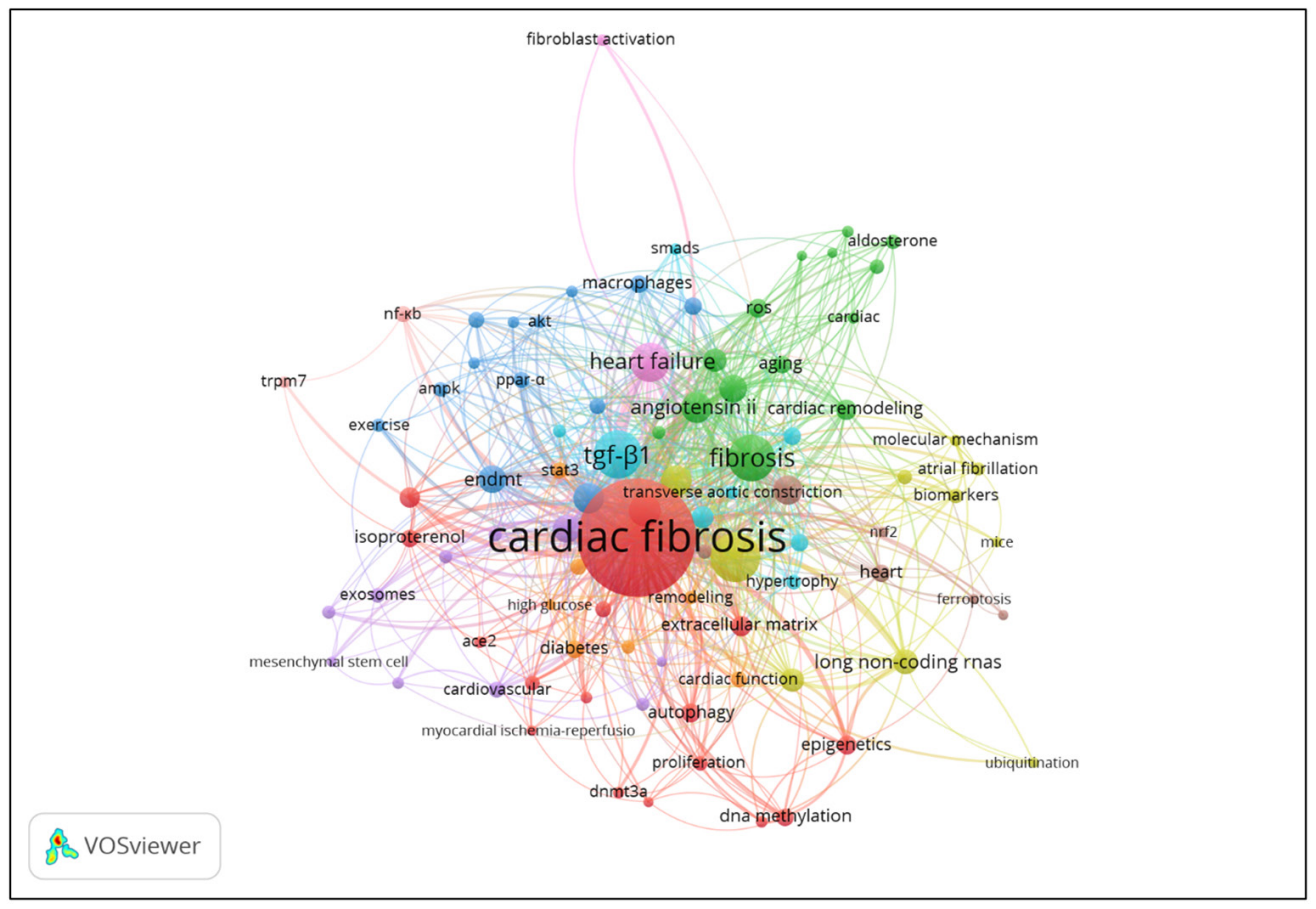
Network visualization of the co-occurrence analysis of the author keywords. This figure maps the relationships between frequently used author keywords in cardiac fibrosis research. Larger nodes represent higher keyword frequency, and color-coded clusters indicate related research themes. Visualization was generated using VOSviewer. Source: Generated by the author(s) using VOSviewer (Van Eck & Waltman^[16]^).

Co-occurrence analysis of author keywords is a bibliometric technique employed to uncover the conceptual structure of a specific research field. Using VOSviewer, this analysis identifies how frequently pairs of keywords appear together in the same publication, revealing relationships between key themes and topics (Fig. 7). In the resulting network, keywords (nodes) that co-occur frequently are positioned closer together and are often grouped into clusters, representing distinct subdomains within the broader research area. This method is instrumental in highlighting emerging trends, interdisciplinary linkages, and the intellectual organization of the literature. The link to the VOSviewer file for the co-occurrence network map is provided in Appendix B.

In this analysis, the full counting method was used, meaning that each co-occurrence of keywords is counted equally, regardless of how often the keyword appears in other contexts. A minimum occurrence threshold of 5 was applied, ensuring that only those keywords that appeared at least 5 times across the dataset were included in the visualization. Out of an initial 1599 unique keywords, 89 keywords met this threshold, forming the basis of the network.

In the visualization (Fig. 7), node size is directly proportional to the frequency of occurrence of a given keyword. Larger nodes (e.g., “cardiac fibrosis,” “fibrosis,” “heart failure”) denote higher prominence and centrality in the literature. Meanwhile, colors represent distinct clusters generated through modularity-based clustering algorithms. Each cluster groups keywords that co-occur more frequently with each other than with those in other clusters, suggesting a coherent thematic area.

Based on the co-occurrence data and clustering algorithm, 10 clusters were identified. Each cluster corresponds to a thematic subfield within the broader context of cardiac fibrosis research.

#### 4.9.1. Cluster 1: Molecular and Epigenetic Mechanisms of Cardiac Fibrosis

This is the largest cluster, encompassing a substantial body of research that focuses on the molecular signaling pathways and epigenetic regulators contributing to cardiac fibrosis. Key themes within this cluster revolve around cellular dysfunction, stress-induced signaling cascades, and gene regulation mechanisms.

First, a significant segment of the literature emphasizes the roles of autophagy, pyroptosis, and mitochondrial dynamics in the pathogenesis of cardiac fibrosis. Studies suggest that the dysregulation of these intracellular processes under stress conditions, such as diabetic cardiomyopathy or myocardial infarction, can trigger maladaptive remodeling (e.g., DNMT3A, mitochondrial fission, and cardiac dysfunction). Second, researchers underscore the importance of epigenetic modifications, including DNA methylation and histone modification, as pivotal mechanisms modulating fibrotic gene expression. Genes such as DNMT3A and pathways related to DNA methylation are increasingly identified as therapeutic targets for reversing fibrosis-associated phenotypes, particularly in the context of diabetic cardiac fibrosis. Third, the studies in this cluster often involve the interaction between pro-fibrotic cytokines and experimental agents, including isoproterenol-induced models, to examine signaling changes in response to pathological stimuli. The roles of high glucose environments or ischemia–reperfusion injury in enhancing fibrotic remodeling through molecular regulators such as angiotensin-converting enzyme 2 and nucleotide-binding domain, leucine-rich-containing family, pyrin domain-containing-3 inflammasome are also analyzed in these works.

Studies within this cluster add to an increased understanding of the cellular and subcellular basis of fibrosis and new targets of intervention through the exploitation of greater understanding of epigenetic control, cell death pathways, and inflammatory responses.

#### 4.9.2. Cluster 2: Hormonal and Oxidative Pathways in Fibrosis Progression

This cluster emphasizes on neurohormonal signal pathways and oxidative stress levels that lead to the remodeling of fibrosis in cardiac tissue.. The most important keywords are angiotensin II and aldosterone, which indicate the key role of the renin–angiotensin–aldosterone system (RAAS) dysregulation.

The first of these themes is the pro-fibrotic nature of RAAS components, in particular, how angiotensin II and aldosterone stimulate fibroblasts and result in the deposition of ECM. The downstream effects of these hormones are also studied using intermediaries like the mineralocorticoid receptor and their pharmacological regulation by pharmacological agents like eplerenone. Second, the cluster brings to focus the role of oxidative stress, which is supported by the often co-occurring variables of reactive oxygen species, nicotinamide adenine dinucleotide phosphate oxidase, and inflammation. Such investigations explain the connection between oxidative injuries and fibrotic signaling pathways to explain mechanisms of cardiac remodeling and stiffness. Third, the literature suggests that age-related and hypertensive changes are vital causes of fibrosis, as it is associated with chronic and old age conditions like hypertension and aging, and their associated molecular alterations in cardiac structure. This cluster adds to the field by describing the hormonal and redox-sensitive modulation of fibrosis, providing insights into the design of antioxidant or RAAS-blocking drugs.

#### 4.9.3. Cluster 3: Metabolic Dysregulation and Inflammatory Remodeling in Diabetes-Associated Fibrosis

This cluster focuses research on metabolic causes of cardiac fibrosis with a specific attention to diabetes, inflammation due to obesity, and immune cell infiltration. First, there is a prevalence of diabetes-related terms, including diabetic cardiomyopathy, diabetes mellitus, and high glucose, denoting the adverse effects of a hyperglycemic milieu, which impairs fibrotic remodeling due to metabolic stress and glyco-toxicosis. These conditions also favor the induction of signaling pathways, including adenosine monophosphate-activated protein kinase, peroxisome proliferator-activated receptor alpha, and protein kinase B. Second, there is significant emphasis on inflammatory agents, which include macrophages, endothelial-to-mesenchymal transition, and apoptotic mechanisms. Such studies have shown that immune cells and vascular dysfunction promote fibrosis through a shift in the cellular phenotype of cardiac cells. Third, exercise and sodium–glucose cotransporter 2 inhibitors as interventions are also assessed as having protective roles in fibrosis and indicate an increased interest in lifestyle and treatment measures to counteract cardiac consequences of diabetes. Overall, this cluster illuminates the multifaceted interplay of metabolic imbalance, immune response, and fibrosis, to offer a foundation of translational research addressing diabetic groups.

#### 4.9.4. Cluster 4: Regulatory RNAs and Genetic Biomarkers in Fibrosis

This cluster focuses on the emerging evidence of the presence of noncoding RNAs and molecular biomarkers in regulating and diagnosing cardiac fibrosis. First, studies focus on lncRNAs, microRNAs, and circular RNAs as post-transcriptional regulators of gene expression. These molecules modulate pathways involved in fibrosis, hypertrophy, and ECM turnover. Second, biomarker discovery, especially using animal models like mice, and disease conditions like myocardial infarction and atrial fibrillation, also has a high interest. Such biomarkers display promise in early detection and personalized intervention. Third, this cluster captures studies into the underlying molecular mechanisms and pathways that are regulated by these RNAs, which provides mechanistic understanding of the mechanisms involved in fibrosis at the gene expression level. The studies in this cluster correspond to the state of the art of molecular diagnostics and RNA therapeutics which together leads toward a precision medicine perspective to cardiac fibrosis.

#### 4.9.5. Cluster 5: Cellular Therapies and Anti-Fibrotic Interventions

Cluster 5 involved cellular strategies and anti-fibrotic molecules targeting fibrosis via modulation or reversal of fibrosis. One of those interesting details is the fact that myofibroblasts, cardiomyocytes, and mesenchymal stem cells stand out as other focal points, with cell-based therapy being one of the major themes. Such cells are investigated in terms of their ability to replace tissue or regulate fibrosis through paracrine signaling. Second, exosomes and extracellular vesicles are given attention, and can provide a means of intercellular communication, with potential therapeutic applications in the delivery of RNA and proteins. Third, natural or synthetically produced bioactive compounds, including curcumin, show anti-inflammatory and anti-fibrotic activities, which means an increasing interest in making use of bioactive compounds. The cluster will advance regenerative medicine and cell therapy solutions to complement molecular-based therapies to treat cardiac fibrosis.

#### 4.9.6. Cluster 6: TGF-β Signaling and Structural Remodeling Pathways

This cluster includes studies focusing on the TGF-β signaling pathway and downstream signaling events that contribute to cardiac fibrosis.

To begin with, the canonical TGF-β1/Smad signaling axis has been actively discussed in the literature, and its central role has been established in fibroblast activation, collagen production, and ECM deposition. Keywords, TGF-β1, Smad3, Smads, and collagen, imply intensive investigation of this pathway as a primary mediator of fibrotic remodeling. Second, experimental models are often used within this cluster to induce mechanical stress, such as transverse aortic constriction and pressure overload, and to determine the fibrogenic response. They are used to model hypertrophic and fibrotic reactions in the face of chronic hemodynamic overload. Third, the implication of matrix metalloproteinases and related ECM regulators class means that the emphasis is on the turnover and remodeling of the matrix, which implies a dual focus on the production and breakdown of the ECM components. Collectively, this cluster has made important contributions to mechanistic knowledge related to fibrotic signaling and remodeling of cardiac tissue, and provides a solid foundation of molecular targets towards developing therapeutics.

#### 4.9.7. Cluster 7: Metabolic Stress and Cardiac Function in Diabetic Contexts

Cluster 7 investigates the effect of metabolic stressors on cardiac performance, and the significant study on diabetes-related cardiac remodeling. To begin with, such major themes as diabetes, high glucose, and diabetic cardiomyopathy depict how authors have focused on the fibrotic changes that occur as a result of hyperglycemia. These studies investigate how prolonged exposure to elevated glucose levels impairs myocardial structure and function. Second, keywords such as cardiac function, cardiomyopathy, and remodeling point to investigations of functional outcomes of fibrotic processes, suggesting a translational focus on how structural changes compromise cardiac performance. In addition to this, signal transduction-related molecules like signal transducer and activator of transcription 3 and relaxin therapies are examined in terms of their effects on limiting fibrotic remodeling, suggesting a molecular-targeted strategy to maintain functionality during metabolic stressors. This cluster highlights the pathophysiological link between diabetes and heart disease, emphasizing the need for integrated metabolic and cardiac care strategies.

#### 4.9.8. Cluster 8: Oxidative Stress and Cell Death Mechanisms in Fibrosis

Cluster 8 centers on the role of oxidative stress and regulated cell death pathways, particularly ferroptosis, in the development of cardiac fibrosis. First, keywords such as oxidative stress, sirtuin 1, and nuclear factor erythroid 2-related factor 2 strongly focus on redox imbalance and the cellular antioxidant response. These studies examine how disrupted oxidative homeostasis promotes fibrosis through fibroblast activation and inflammation. Second, the emergence of ferroptosis, a regulated iron-dependent form of cell death, suggests growing interest in novel cell death pathways and their implications for myocardial injury and fibrotic progression. Third, gut microbiota appears in this cluster, reflecting an emerging area of research investigating the gut–heart axis and how microbial metabolites influence oxidative stress and cardiac inflammation. Research in this cluster contributes to an evolving understanding of redox biology in cardiovascular disease, presenting new targets for antioxidant and anti-ferroptotic therapies to prevent fibrosis.

#### 4.9.9. Cluster 9: Clinical Outcomes and Fibroblast Biology

This cluster primarily focuses on the clinical manifestations of cardiac fibrosis and fibroblast-mediated mechanisms underlying disease progression. Heart failure emerges as a dominant theme, positioning fibrosis as a key contributor to impaired cardiac output and end-stage cardiovascular disease. These studies emphasize the translational relevance of fibrosis research to clinical cardiology. Second, fibroblast activation is a central concept, with literature exploring the mechanisms that drive the transformation of fibroblasts into myofibroblasts, a process critical to collagen deposition and tissue stiffening. Studies done in this cluster have significant observations concerning the gap between molecular pathology and clinical outcomes, supporting the significance of fibroblasts as therapeutic targets and biomarkers in managing heart failure.

#### 4.9.10. Cluster 10: Inflammation and Ion Channel Regulation

The last cluster revolves around pro-inflammatory signaling and regulation of ion channels relative to cardiac fibrosis. First, nuclear factor kappa-light-chain-enhancer of activated B cells, as a master regulator of inflammatory gene transcription, is mentioned due to its central role in the enhancement of cytokines and activation of fibroblasts. Its implication highlights the significance of inflammation as a fibrotic progression. Second, transient receptor potential melastatin 7, a transient receptor potential ion channel, appears in conjunction with nuclear factor kappa-light-chain-enhancer of activated B cells, suggesting a novel mechanistic link between ion flux, calcium signaling, and inflammatory activation in fibrotic heart disease. Although smaller than other clusters, this group reveals a specialized and emerging area of fibrosis research focused on the crosstalk between electrophysiology and inflammation, potentially uncovering new avenues for anti-inflammatory and anti-arrhythmic therapies.

The co-occurrence network analysis significantly contributes to understanding the multifaceted landscape of cardiac fibrosis research. It maps the intellectual structure of the field, revealing how subdomains such as TGF-β signaling, epigenetic regulation, noncoding RNAs, and metabolic syndrome interrelate. Then, it identifies emerging trends (e.g., ferroptosis, exosomes, gut microbiota) that may shape future research trajectories.

In addition, it highlights translational potential by bridging basic mechanisms (e.g., oxidative stress, fibroblast activation) with clinical implications (e.g., heart failure, biomarker development). Interestingly, it also assists scholars and clinicians in identifying research gaps, collaborative opportunities, and interdisciplinary linkages, especially those between molecular biology, cardiology, and regenerative medicine.

## 5. Discussion

### 5.1. Summary of key findings

To the best of our knowledge, this is the first bibliometric analysis on cardiac fibrosis in the scope of Asia and the Oceania continent. This bibliometric analysis offers a comprehensive mapping of cardiac fibrosis research across the mainland from 1985 to 2025, revealing significant developments in the field’s evolution, productivity, and intellectual structure. A total of 983 publications were identified, with a marked increase in output observed particularly from 2010 onwards, reaching its zenith in 2024. China emerged as the most prolific contributor, accounting for 76.2% of total publications and leading in both total citations (TC = 19,772) and *h*-index (*h* = 63), underscoring its dominant role in advancing this research domain. Although contributing fewer articles, Australia demonstrated the highest citation per publication (C/P = 62.05), suggesting a high degree of scientific influence and impact per research output.

Original research articles constituted the majority (86.98%), indicating a robust foundation of empirical investigations. Journals such as PLOS ONE, Scientific Reports, and Frontiers in Pharmacology were among the most active platforms, reflecting cardiac fibrosis studies’ interdisciplinary and translational nature. Co-authorship and keyword analyses further revealed strong collaborative networks and emerging thematic foci, particularly around signaling pathways, molecular targets (e.g., TGF-β, Smad, noncoding RNAs), and fibrosis-related pathophysiological mechanisms.

### 5.2. Interpretation of findings

The escalating publication volume in the past decade aligns with growing clinical and scientific recognition of cardiac fibrosis as a central contributor to heart failure and other chronic cardiovascular pathologies. The predominance of molecular and cellular keywords such as “macrophage,” “extracellular matrix,” and “inflammation” suggests a sustained research emphasis on elucidating fibrotic mechanisms at the microscopic level. This trend reflects broader global efforts to shift from phenotypic characterization to targeted molecular diagnostics and interventions.

China’s prolific output likely reflects national investment in cardiovascular science and a strategic emphasis on translational medicine. Traditional Chinese medicine (TCM) is deeply rooted in the national health system as well as in the education sector, where it has been practiced as a system of medicine parallel to Western medicine in the country. This institutionalized dual training helps clinicians and medical students become competent in training both in TCM and the Western medical systems to support a more holistic patient-centered approach.^[38]^ Furthermore, the government of China actively supports the development of TCM by providing significant funds for research and strategic policy measures. Specifically, the 14th Five-Year Plan focuses on increasing the number of integrated diagnostic and therapeutic frameworks, reaffirming the determination by the state to unite the practice of traditional and modern approaches to medical care.^[39]^ Moreover, this combination, which could be compared with the embodiment of “Chinese characteristics,” represents a very conscious and logical attempt to reconcile traditional Chinese medical practices with modern biomedical breakthroughs. Consequently, China’s medical education and healthcare delivery systems offer a unique example of combining the strengths of both Western and TCM, with one benefiting the other in a complementary and coordinated fashion.

However, other countries, such as Australia, have focused more on quality research, albeit with lower publication numbers indicated by increased citation metrics. This duality implies that volume of activity is a significant measure of scientific activity and involvement, but that impact and innovation tend to be highly concentrated within a few institutions and research agglomerations. This difference between quantity and quality highlights a very important split in the modern bibliometric judgment. High-impact research usually appears in organizations that rely heavily on methodological robustness, cross-disciplinary cooperation, and publication in high-profile journals. Examples of this model are the Baker Heart and Diabetes Institute and Monash University, top research centers in Australia that conduct research that attracts international attention and makes a significant contribution to cardiovascular and fibrosis research.^[40]^ Thus, the Australian example shows the need to increase the quantity of research combined with strategic emphasis on quality and translational importance.

The relevance of the pharmacological journals and the prevalence of drug-related keywords prove that there is an interest in the therapeutic manipulation of fibrosis. This pattern is consistent with the growing attention to identifying anti-fibrotic compounds, which can also prevent or reverse the progression of myocardial remodeling processes. Preclinical and early clinical research results powerfully support such urgency. In 1 instance, high-throughput screening identified salinomycin as a potent fibroblast activation and ECM deposition inhibitor that successfully prevented myocardial remodeling in mouse models.^[41]^ McKinsey et al^[42]^ review existing epigenetic therapies aimed at heart failure, maladaptive molecular pathways focusing on noncoding RNA-based compounds in early-stage clinical development to modulate cardiac remodeling and fibrosis. Furthermore, the increasing popularity of journals with an interdisciplinary focus implies that the study of cardiac fibrosis is becoming more open to collaboration with other fields such as regenerative medicine, molecular biology, or bioengineering. Perrucci et al^[43]^ give insights into cardiac fibrosis’s molecular and cellular pathogenesis, especially regarding integrins and ADAMTS proteins as novel candidates to target remodeling of the ECM, facilitating regeneration and therapeutic approaches. This represents an intersection of molecular biology with regenerative aims.

### 5.3. Implications for practice

This current study has strategic significance in developing cardiac fibrosis research and promoting it in clinical innovation. Identifying high-impact authors and institutions can be a map toward developing research collaborations, training formats, and funding distributions. An example is developing the networks between new Southeast Asian centers and the existing institutions in Australia or Japan, potentially contributing to regional capacity building and knowledge sharing.

Second, the emphasis on molecular mechanisms in recent studies suggests a maturing field increasingly poised for translational breakthroughs. Clinicians and policymakers should take note of the expanding preclinical evidence base for anti-fibrotic therapies, and support early-phase clinical trials targeting key pathways such as TGF-β, renin-angiotensin, and epigenetic regulators.

Third, the limited representation of clinical trials and health systems research in the literature indicates an opportunity for integrative studies that bridge laboratory findings with patient outcomes. Real-world data integration, longitudinal cohort studies, and biomarker validation studies are essential for contextualizing preclinical discoveries and guiding evidence-based practice.

### 5.4. Recommendations for future research

Several directions for future research emerge from the current findings. First, enhanced transdisciplinary integration. Research should increasingly incorporate computational biology, systems medicine, and bioinformatics insights to model complex fibrotic networks and identify novel therapeutic targets. Second, a greater focus on translational research. Bridging the gap between bench and bedside remains a critical need. Future studies should prioritize in vivo validation, clinical translation, and multi-center trials aimed at evaluating candidate anti-fibrotic agents. Third, expansion of collaborative networks. Despite visible collaboration patterns, there is a need to encourage cross-national and cross-disciplinary partnerships, particularly involving South and Southeast Asian underrepresented countries. Fourth, the development of standardized metrics. Establishing harmonized criteria for measuring fibrotic burden, both in preclinical and clinical settings, would enhance comparability and replicability of findings across studies. Fifth, inclusion of emerging research themes. Novel areas such as the gut–heart axis, immunometabolism, cardiac fibroblast heterogeneity, and machine learning in histopathological analysis deserve greater bibliometric and empirical attention.

### 5.5. Limitations and future direction

This study is subject to limitations inherent to bibliometric analyses. The citation-based metrics inherently favor older publications and may not accurately reflect the impact of more recent articles. Nevertheless, the current analysis provides a foundational understanding of research dynamics in cardiac fibrosis and offers a data-driven basis for shaping future research strategies. To overcome the identified limitations, future bibliometric studies should consider employing multiple databases, author identifier systems (e.g., ORCID), and altmetric indicators to capture broader dimensions of research impact.

## 6. Conclusions

This bibliometric study aims to comprehensively map the historical context of cardiac fibrosis research in Asia and Oceania during the last 4 decades (1985–2025). Its main aims were to evaluate research productivity, identify influential authors and institutions, elucidate thematic trends, and explore collaboration networks within this domain. Using data obtained through the Scopus database, the study aimed to identify answers to key questions on the evolution, influence, and trend of research on cardiac fibrosis in the regions of interest.

The analysis shows that the output of cardiac fibrosis research has significantly grown in recent decades in terms of academic interest and investment in studying cardiac fibrosis. China was the main contributor based on the quantity as well as cumulative contributions, and Australia had the greatest citation impact per publication. Important participants in the collaborative network were high-output institutions, including Harbin Medical University and the Baker Heart and Diabetes Institute. The focus of research has evolved over time to include more molecular details with frequent focus on TGF-β, Smad pathways, and noncoding RNAs, and highlight the advances, sophistication, and translational promise of new research.

This study contributes to the field by providing a quantitative and visualized overview of cardiac fibrosis research trends, facilitating a deeper understanding of its intellectual structure and developmental trajectory. It extends existing bibliometric literature by focusing specifically on the Asia and Oceania regions of growing scientific prominence that have not been comprehensively examined in prior analyses. The findings serve as a valuable resource for scholars, funding agencies, and policymakers by identifying influential contributors, high-impact publication venues, and emerging research frontiers.

From a practical standpoint, the identification of dominant research themes and prolific institutions may guide strategic collaborations and inform regional and international policy decisions related to research funding and academic partnerships. Furthermore, the increasing concentration on molecular and pharmacological studies underscores the field’s readiness to transition from mechanistic discovery to therapeutic development.

Nevertheless, there are limitations to this study. Temporal bias can be an issue when using citation-based metrics over more recent publications. To get a broader view of the field, future studies are encouraged to be conducted on multi-database, altmetric indicators, and machine learning-based content classification. A deeper search within the system to locate latent thematic trends as an expansion of the keyword string might also be used to enhance the analytical framework.

To summarize, this bibliometric study emphasizes the increasing scientific impetus of the study of cardiac fibrosis research in Asia and Oceania, and adds importance to local contributions to the progress in cardiovascular science globally. This study validates the importance of using bibliometric methods to explain the development of complex biomedical fields and supports future processes aimed at providing those analyses to be used by strategic planning, funding, and research policy. By outlining key actors, collaborative dynamics, and thematic priorities, this study provides the basis for future research and interdisciplinary developments in cardiac fibrosis.

## Acknowledgments

The study was a project outcome of the Fundamental Research Grant Scheme (FRGS) (Project number: FRGS/1/2021/SKK0/UITM/03/2) from the Ministry of Higher Education, Malaysia, with RMC no: 600-RMC/FRGS 5/3 (064/2021). We want to acknowledge the support given by the Faculty of Medicine, Universiti Teknologi MARA, in producing this manuscript. The authors acknowledge the use of ChatGPT (https://chatgpt.com/) and Grammarly (https://app.grammarly.com/) for enhancing the readability and accuracy of this work. ChatGPT was used to improve the readability and clarity of the text, while Grammarly was used to correct the spelling errors. While the authors acknowledge the usage of AI, they maintain that they are the sole authors of this article and take full responsibility for the content therein, as outlined in COPE recommendations.

## Author contribution

**Conceptualization:** Thuhairah Hasrah Abdul Rahman, Vimala RMT Balasubramaniam, Nurdiyana Nasrudin, Nasibah Azme.

**Data curation:** Arifah Ahmad Damahuri, Nasibah Azme.

**Investigation:** Arifah Ahmad Damahuri.

**Methodology:** Arifah Ahmad Damahuri, Maslinawati Mohamad, Nasibah Azme.

**Software:** Arifah Ahmad Damahuri, Maslinawati Mohamad.

**Supervision:** Maslinawati Mohamad, Thuhairah Hasrah Abdul Rahman, Vimala RMT Balasubramaniam, Nurdiyana Nasrudin, Nasibah Azme.

**Validation:** Maslinawati Mohamad, Thuhairah Hasrah Abdul Rahman, Vimala RMT Balasubramaniam, Nasibah Azme.

**Writing – original draft:** Arifah Ahmad Damahuri, Nasibah Azme.

**Writing – review & editing:** Maslinawati Mohamad, Vimala RMT Balasubramaniam, Nurdiyana Nasrudin, Nasibah Azme.




