# Supplementary material for: The heart’s fibrous web: A bibliometric analysis of cardiac fibrosis in Asia and Oceania
Source: Medicine (Baltimore). 2026 May 29;105(22):e49017. doi: 10.1097/MD.0000000000049017 (PMC13225514; doi:10.1097/MD.0000000000049017)
Supplement: Supplementary file 1 [file medi-105-e49017-s001.docx]

**Appendix A. List of Countries Included under Asia and Oceania Based on the United Nations M49 Geoscheme**

data from https://unstats.un.org/unsd/methodology/m49/

| **Region** | **Sub-region** | **Countries and Territories** |
| --- | --- | --- |
| Asia | Central Asia | Kazakhstan, Kyrgyzstan, Tajikistan, Turkmenistan, Uzbekistan |
|  | Eastern Asia | China, China - Hong Kong Special Administrative Region, China - Macao Special Administrative Region, Democratic People's Republic of Korea, Japan, Mongolia, Republic  of Korea |
|  | Southeastern Asia | Brunei Darussalam, Cambodia, Indonesia, Lao People's Democratic Republic, Malaysia,  Myanmar, Philippines, Singapor e, Thailand, Timor-Leste, Viet Nam |
|  | Southern Asia | Afghanistan, Bangladesh, Bhutan, India, Iran, (Islamic Republic of), Maldives, Nepal,  Pakistan, Sri Lanka |
|  | Western Asia | Armenia, Azerbaijan, Bahrain, Cyprus, Georgia, Iraq, Israel, Jordan, Kuwait, Lebanon, Oman, Qatar, Saudi Arabia, State of Palestine, Syrian Arab Republic, Turkey, United Arab  Emirates, Yemen |
| Oceania | Australia and New  Zealand | Australia, Christmas Island, Cocos (Keeling) Islands, Heard Island and McDonald Islands,  New Zealand, Norfolk Island |
|  | Melanesia | Fiji, New Caledonia, Papua New Guinea, Solomon Islands, Vanuatu |
|  | Micronesia | Guam, Kiribati, Marshall Islands, Micronesia (Federated States of), Nauru, Northern  Mariana Islands, Palau, United States Minor Outlying Islands |
