# Supplementary material for: The heart’s fibrous web: A bibliometric analysis of cardiac fibrosis in Asia and Oceania
Source: Medicine (Baltimore). 2026 May 29;105(22):e49017. doi: 10.1097/MD.0000000000049017 (PMC13225514; doi:10.1097/MD.0000000000049017)
Supplement: Supplementary file 2 [file medi-105-e49017-s002.docx]

**Appendix B. Visualizations of Bibliometric Analysis**

This appendix provides access to the VOSviewer files used in the study. The first link and QR code lead to the author co-authorship density visualization, while the second corresponds to the author keyword co-occurrence network. These supplementary materials allow readers to explore the underlying bibliometric data and relationships, supporting reproducibility and broader use of the analysis.

| **Co-authorship (Density visualization) - Authors** | |
| --- | --- |
| LINK | QR CODE |
| <https://tinyurl.com/26q58ym3> | 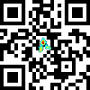 |
| **Co-occurrence (Network visualization) – Author keywords** | |
| LINK | QR CODE |
| <https://tinyurl.com/27qvjg7t> | 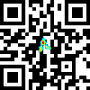 |
